# The Unobserved Heterogeneneous Influence of Gamification and Novelty-Seeking Traits on Consumers’ Repurchase Intention in the Omnichannel Retailing

**DOI:** 10.3389/fpsyg.2020.01664

**Published:** 2020-07-10

**Authors:** Cheong Kim, Francis Joseph Costello, Kun Chang Lee

**Affiliations:** ^1^SKK Business School, Sungkyunkwan University, Seoul, South Korea; ^2^Economics Department, Airports Council International (ACI) World, Montreal, QC, Canada; ^3^Department of Health Sciences and Technology, SAIHST (Samsung Advanced Institute for Health Sciences and Technology), Sungkyunkwan University, Seoul, South Korea

**Keywords:** omnichannel, gamification, novelty-seeking, unobserved heterogeneity, repurchase intention

## Abstract

As information technology continues to provide a platform for any business willing to engage in diverse channels, it has ushered in a continuous evolution of ways to attract and maintain a given customer base. One of the latest trends seen in the retailing industry is the implementation of an omnichannel business strategy. As a result, the number of businesses now implementing such a strategy has led to a lack of differentiation amongst competitors. Therefore, it is no surprise that omnichannel retailers have had to rethink and acquire a new competitive advantage through the exploration of new and innovative strategic activities. Prior work on services has shown gamification to be a successful strategy in enhancing customer loyalty, promoting positive word-of-mouth, and enhancing greater engagement with the offered service. Also, offering hedonic values (one of gamification’s main premises) has been an effective strategy for engaging customers as well as promoting repeat purchase intentions. Despite this, the potential effects of gamification within an omnichannel setting is not yet explored, and thus the rationale of this study. In exploring this gap, we employed means-end chain theory as a basis in which to discover the potential of gamification. Additionally, as gamification is a novel method in omnichannel research, this paper strived to explore the moderating effect of the novelty-seeking traits and unobserved heterogeneous behaviors of consumers. This research was based on 440 valid questionnaires in a survey dataset from Amazon M-Turk. The results provided strong evidence of the underlying proposition within the research models. Specifically, this study showed that gamification could be a potential unique feature used for engaging consumers onto one’s platform, especially consumers with a novelty-seeking trait. We did, however, find that this finding does not apply to the case for all consumers. Therefore, the implication of this research suggests to practitioners that its implementation should be approached through an opt-in rather than a compulsory option.

## Introduction

With the ever-expanded capabilities of computer-mediated technologies, many companies have adapted their retailing to fit the instant gratification expected from younger consumers. Through the integration of multiple channels, omnichannel retailing provides a solution to allow the omni-channel consumer (OCC) to seemingly shop offline, online, through mobile, and/or social commerce (Yurova et al., 2017). To compete with mega-sized firms such as Amazon and eBay, omnichannel retailing ventures have had to acquire a competitive advantage whereby scale is replaced with computer-mediated intelligence as the primary strategy. For a couple of decades now, the prevailing logic in retailing research has seen utilitarian and hedonic values investigated extensively, with utilitarian value shown to be more dominant in users’ purchasing behavior (i.e., Overby and Lee, 2006; Chiu et al., 2014). In addition to utilitarian value, hedonic value is also considered a vital component of business strategy (Arnold and Reynolds, 2003; Overby and Lee, 2006; To et al., 2007; Park and Ha, 2016; Dedeoglu et al., 2018). Outside of retailing, a recent trend in business (Suh et al., 2017) and services have shown gamified experiences (ones directly linked to hedonic usage patterns) attempt to convert utilitarian mechanics into more hedonically oriented ones (Hamari, 2013). As highlighted by Yurova et al., OCCs can fluctuate between utilitarian and hedonic retailing patterns depending on the product, available information and channel being used (Yurova et al., 2017). With utilitarian values well-established, attempts to delve deeper into the aspects of hedonic usage patterns that OCCs face, could be of great value.

Scholars argue that hedonic values are based on consumers’ pleasure and happiness and could be described as the chief good in life (Van der Heijden, 2004). Prior research suggests that utilizing hedonic values is an effective strategy for engaging customers (Overby and Lee, 2006), as well as promoting repeat purchase intentions (Chiu et al., 2014). However, in contrast to these well-known theories, omnichannel research has thus far shown that hedonic motivations are not a cause for omnichannel purchase intention (Juaneda-Ayensa et al., 2016). According to Juaneda-Ayensa et al. (2016), hedonic motivation, defined as the fun or pleasure achieved from interacting with a technology (Brown and Venkatesh, 2005), is found not to be a motivating factor in its present form. As omnichannel retailing is premised on the use of technology (Herhausen et al., 2015), understanding the potential ways this can be reversed so that OCCs can feel fun or pleasure using the technology calls for an alternative approach.

We propose an exploration of gamification. Gamification in business and services has been explored and successfully implemented through game components within non-game contexts (Hamari and Koivisto, 2015). Based on this rationale, we hypothesize that gamified hedonic dynamics could also potentially be explored within an omnichannel setting to significant effect (Hamari, 2013). Through fun, play, badge, and goal-setting dynamics, gamification extends the entertaining features of games into real-world applications (Hamari, 2013; Hwang and Choi, 2019) and is increasingly being embraced by professionals (Hwang and Choi, 2019). Thus, our exploration attempts to explore how gamification could fit within an OCCs shopping patterns especially embedded within the novel use of technology within omnichannel retailing (Juaneda-Ayensa et al., 2016). In order to explore gamification within an omnichannel setting, we propose the following research questions as a way to lead the objectives of this study: (1) Is gamification a viable tool for repeat purchase intention within the setting of omnichannel retailing? (2) To what extent does novelty-seeking have an influence on omnichannel consumers’ repurchase intention? (3) Do the relationships of hedonic value, gamification, repeat purchase intention, and novelty-seeking differ when unobserved heterogeneity is introduced within the sample?

In order to answer these research questions, this study adopted means-end chain theory as a theoretical basis in which to reveal the potential of gamification that could have an influential mediation role in connection with the hedonic value and repurchase intention of OCCs. Furthermore, as gamification is presented as a novel strategical approach in omnichannel research, this study attempted to discover the moderating effect of the novelty-seeking characteristics and unobserved heterogeneous behaviors of consumers.

The rest of the paper is organized as follows. The second chapter reviews the theoretical background and develops the hypotheses of this research. The third chapter proposes the research methodology, and the next chapter summarizes the results and the implications. Finally, the last chapter includes conclusions, limitations, and suggested topics for future study.

## Theoretical Background and Hypotheses

Through the lens of means-end-chain (MEC) theory, consumers can be seen to have three levels of cognitive abstraction—attributes, consequences, and values—that provide them with a guide to their shopping behavior (Gutman, 1982). These link the purchasing behaviors of consumers with perceived value, which, in turn, increases the likelihood of a repurchase intention (Chiu et al., 2014). Attributes are seen as physical and concrete factors that a service or product provides, with consequences, good or bad, being the primary reactionary outcome of this consumption (Gutman, 1982). Originally presented as a hierarchy of goals, Chiu et al. (2014) explain that when it comes to the retailing context, consumers acquire a product mostly due to the values gained-which is the last link in the MEC theory-not its attributes *per se*. We follow a similar line of reasoning and focus on these two original features. Specific to this paper, we focus on the previous link in the MEC chain using hedonic values with the inclusion of gamification. We use this to explain how consumers obtain their values once they believe the purchase has helped in achieving the higher-level goals that could be interpreted as values through lower-level goals that could be construed as benefits (Gutman, 1982; Chiu et al., 2014). In fact, for two decades, marketers have seen value as one of the leading causes of repurchase intention (Neal, 1999); thus, we place gamification as a potential value proposition that provides extra cost to consumers. Therefore, the value within the MEC theory provides an underlying principle and direction of this current study.

### Omnichannel Retailing

In the traditional context of retailing, offline brick and mortar stores were the only possible choice for consumers. However, with the remarkable evolution of information communication technology, digitalization in shopping, including website commerce, mobile commerce, and social commerce, have become a formidable archon in the global retailing industry (Verhoef et al., 2015; Juaneda-Ayensa et al., 2016). For consumers, digital retailers provide excellent benefits, such as no queues, simplified price comparisons, diverse choices, remote store access, and instantaneous digital purchases (Niranjanamurthy et al., 2013). In addition to consumer benefits, digital channels also provide novel opportunities to retailers such as saving upfront costs due to no need for physical stores, 24-h operation, labor cost reductions, and unlimited shelf space (Niranjanamurthy et al., 2013). Because of these advantages from digital retailing, offline-origin retailers (i.e., Homedepot, Bestbuy, Costco) have penetrated into the digital setting in order to acquire competitiveness. This has led to an integration of online and offline distribution channels (Herhausen et al., 2015). Furthermore, online-origin retailers such as Amazon, eBay, and Alibaba have also joined the arena for competing in the era of online retail (Brynjolfsson et al., 2013; Bell et al., 2014; Piotrowicz and Cuthbertson, 2014; Beck and Rygl, 2015).

Nevertheless, this initial introduction of channel integration could not entirely fulfill the consumer needs of consistency, uniform, and integration for the products and services. The initial business strategy of multichannel retailers was to provide different goods depending on the channel (Cook, 2014; Piotrowicz and Cuthbertson, 2014; Juaneda-Ayensa et al., 2016). However, this did not meet the demands of younger consumers whose lives are changing so fast that adapting channels to them was no longer a feasible proposition (Verhoef et al., 2015). In order to fill this gap in consumer perspectives and provide a seamless and unwavering purchasing experience, retailing firms shifted their attention from multichannel to omnichannel retailing (Verhoef et al., 2015; Lemon and Verhoef, 2016). The overarching philosophy of omnichannel retailing can be explained using a holistic conceptual framework consisting of a three-dimensional pillar that Saghiri et al. (2017) proposed: channel stage, channel type, and channel agent. In his analogy, channel stage represents the value-adding journey, channel type indicates diverse approaches to provide products and services, and channel agent describes management entity of the channel. As many retailing corporations have already turned their strategy to the omnichannel context, only the physical formulation of this conceptual framework of omnichannel from Saghiri et al. (2017) would not guarantee any sales increase without the understanding of consumer traits and competitive business strategy as suggested by Lemon and Verhoef (2016).

Thus far, research in omnichannel retail has presented some diverse and interesting findings. Prior work has shown that omnichannel firms might establish redundant self-created cannibalization and competition in the market that could negatively influence their business sustainability (Kim and Chun, 2018; Luo et al., 2020). It has also been shown that consumers living close to the store are more likely to increase offline spending once induced to buy online by 47% (Luo et al., 2020). Also, the service quality of an internet store has been seen to be a contributing factor to increased satisfaction of an omnichannel retailer (Herhausen et al., 2015). Yurova et al. (2017) revealed the impact of product type (i.e., utilitarian and hedonic products) on omnichannel consumers, while Juaneda-Ayensa et al. (2016) argued that personal innovativeness, effort expectancy, and performance expectancy were the significant influence on the consumers’ purchase intention. Rodríguez-Torrico et al. (2017) discovered that impulsive consumer traits had a strong effect on omnichannel consumers, with Gu and Tayi (2017) suggesting pseudo-showrooming as a business strategy in the omnichannel context. Peltola et al. (2015) indicated organizational culture, price scheme, operation, and consumer interaction of firms were critical components for a successful omnichannel business, and Saghiri et al. (2017) implied integration (i.e., integration in promotion, transaction, pricing, order fulfillment, reverse logistics, product information, and consumer service) and visibility (i.e., visibility in the product, demand, order/payment, stock, delivery, supply) as two essential components of an omnichannel business strategy.

Current insights show that retailers implementing an omnichannel strategy have a lot of aspects to consider when designing for an omnichannel, yet some aspects remain unclear. For example, findings of Juaneda-Ayensa et al. (2016) that hedonic motivation does not have an effect on an OCC seems to go counter to much prior literature on hedonic value within retail (i.e., Overby and Lee, 2006; Chiu et al., 2014). In addition, no prior work has identified the potential behavioral aspect of novelty-seeking. Thus, we link these aspects in line with the investigation of gamification as a potential novel business strategy to be used for promoting hedonic values for OCCs. Further, due to counter narrative presented by Juaneda-Ayensa et al. (2016), we wanted to explore the potential hidden heterogeneous traits of consumers. It might be true that certain consumers sway more to one type of behavioral trait and thus identifying these might help in further understanding what consumers want in their omnichannel experience (Juaneda-Ayensa et al., 2016). Lastly, in line with Juaneda-Ayensa et al. (2016), repurchase intention acted as our dependent variable in order for us to gauge the acceptance of a gamified system within the context of an omnichannel retailer’s setting.

### Repurchase Intention

This paper uses repurchase intention as the central construct for judging the viability of gamification within omnichannel setting. As gamification is a tool that aims to keep people engaged through hedonic-like features such as fun and entertainment (Huotari and Hamari, 2012; Hamari, 2013; Hamari et al., 2014; Hamari and Koivisto, 2015), repurchase intention from consumers who have come back to the website acts as a more realistic measure. This phenomenon is because repeat (i.e., experienced) customers are well versed at comprehending and assessing any information and changes in attributes of omnichannel stores. This is mainly due to the prior experience they have built up with the seller through more than one transaction (Chiu et al., 2014).

Interestingly, more recent literature has found that website identification, which includes service attractiveness, is a vital driver of the repurchase intention of a consumer (King et al., 2016). Service attractiveness helps to shape consumers’ perceptions of value and ultimately helps to improve customer loyalty (Fassnacht and Koese, 2006). Companies like Amazon, in recent times, have successfully implemented a low-pricing strategy and used other incentives to attract new omnichannel shoppers. However, merely attracting more customers on the basis of price may not be a sufficient and sustained competitive strategy when perpetual increases in scale are not viable (Fassnacht and Koese, 2006; King et al., 2016). When formulating a new omnichannel strategy (such as the inclusion of a gamified service element), omnichannel retailers will benefit from deliberately conceptualizing and developing a hedonically pleasing website identity for omnichannel retailers to establish and attract long-term customers, and more importantly, attract repurchase intention (King et al., 2016).

### Hedonic Value

Hedonic value has been considered one of the main influencing attributes on a repurchase intention (Fang et al., 2011; Chiu et al., 2014). Hedonic value is the overall assessment of experiential benefits (i.e., entertainment) a consumer can have during the shopping process (Overby and Lee, 2006). Enjoyment from hedonic value could become a fundamental requisite that motivates fulfillment of shopping activities; thus, a hedonic system should be identified as the level of fun one person has in its use (Van der Heijden, 2004). As suggested by Yurova et al. (2017), OCCs that mostly shop for hedonic products (which can also apply to the entire retail experience) are unlikely to enter engage with a retail store unless they feel it includes an enjoyable shopping channel. Hedonic value suggests that consumers, including OCCs look for entertainment that allows for out-of-routine experiences (Overby and Lee, 2006). Henceforth, consumers use these experiences during the purchase process to rid of any fatigue (McGuire, 1974). In this research, we have used the hedonic value items suggested by Arnold and Reynolds (2003), based on research by McGuire (1974), which has been previously adopted in the context of retail (To et al., 2007; O’Brien, 2010; Chiu et al., 2014).

Although gamification has not been suggested in previous omnichannel research papers, we present the following reasons as to why it should be linked with hedonic values as a separate, yet directly linked, construct. Omnichannel retail, like most systems, has multiple motives in its approach. Nonetheless at its heart is technology. Prior research has consistently shown the prominent effect of hedonic value on the intention to use a technology (Escobar-Rodríguez and Carvajal-Trujillo, 2014; Juaneda-Ayensa et al., 2016). Thus, linking these distinct notions has valid grounds. More recent research has started to suggest that including game-like or hedonic elements, even in systems that seem inappropriate, where game-like features are traditionally extrinsic in nature, can improve a user’s experience and continuance (Lowry et al., 2015). This is believed to be because consumers are more receptive to personalized systems, and thus user-specific data have been utilized to create models for clusters of users. In this vein, a gamified omnichannel system that utilizes hedonic values, as well as user-specific cluster models to target potential OCCs, might help to increase personalization and thus potentially increase repeat custom (Liu et al., 2017). To explore customization, we later implement the clustering of potential users that look for novelty-seeking through unobserved heterogeneity analysis (see sections “Gamification” and “Novelty-Seeking” for more details). Based on the prior theoretical notion of hedonic values and their link with gamification, we propose the following hypotheses in the context of omnichannel:

H1: Hedonic value influences repurchase intention positively in the context of omnichannel.
H2: Gamification influences hedonic value positively.

### Gamification

In recent times, the hype surrounding the concept of gamification has steadily matured (Huotari and Hamari, 2012; Hamari, 2013; Hamari and Koivisto, 2015; Hsu and Chen, 2018). Gamification is now a trending topic within marketing for supporting user engagement and enhancing positive patterns (Hamari et al., 2014). Based on a game or motivational affordances, gamification has been shown to increase user activity, social interaction, quality, and productivity of actions (Hamari, 2013; Hamari et al., 2014). Based on positively induced and innately motivating “gameful” experiences, gamification has high promise in many applications (Huotari and Hamari, 2012).

Embedded within gamification, elements such as points, leader boards, achievements, clear goals, and feedback are all present (Suh et al., 2017; Hsu and Chen, 2018). This means goal setting is one of the critical functions of gamification. Additionally, social benefits through communities and social interaction allow for users of a gamified experience to use social comparison as a tool to know their own standing as well as others (Hamari, 2013). As proclaimed by Hamari (2013), social comparison and goal setting form the basis of gamification when presented through gamified badges. Badges within gamification present gamification in its most basic form, especially when it comes to social comparison and goal setting (Hamari, 2013). For this reason, we explored these two constructs within the environment of omnichannel.

As gamification allows people to compare points and badges, it has persuasive power upon people engaged in its use. This ability to benchmark one’s score remains one of the main rationales of gamification use (Hamari, 2013). Prior research has shown that humans have an innate desire to compare their ability with other humans, known as a “level of aspiration.” In the absence of comparison with other persons, humans have an inability to evaluate their own abilities (Gardner, 1939). Based on this principle, social comparison theory helps to explain the method in which humans overcome this inability and can be seen as the basis for understanding why humans look for comparisons in fellow human beings (Festinger, 1954). The social influence and recognition that one can obtain through a gamified service can help to explain the adoption of this service over other services (Hamari, 2013; Hamari and Koivisto, 2015).

As gamification is derived from games, it is inherently a goal-oriented activity that is seen as a promising area within the literature (Hamari, 2013; Fortes Tondello et al., 2018). Goal-setting theory is based on the principle of motivation and attempts to explain the causes of people’s performance in various tasks (Locke and Latham, 2002). When it comes to goal setting, the content of the goal or its bodily action and intensity, i.e., the difficulty or amount of effort required to complete the given target, are relevant factors to consider. Goal-setting interventions have been found to be powerful motivational interventions and are deemed adequate across many situations and tasks (Landers et al., 2017). A gamified omnichannel system that implements goal setting could be based on many various implementations. However, there exist two common strategies: goals that users can follow or allowing users to set their own individual goals (Fortes Tondello et al., 2018). Goals within gamification can be explicit and identified as quests or objectives of the given problem/target and implicit in the way in which they are presented or pursued, such as earning badges or achievements (Hamari, 2013; McDaniel and Fanfarelli, 2016). Thus, within this paper, we explore the use of goal setting and social comparison that uses badges and levels as a reward as opposed to a financial compensation.

H3: Gamification influences repurchase intention positively in the context of omnichannel.

### Novelty-Seeking

Novelty-seeking (NS) can be defined as a trait that is inherent in consumers and is related to the exploration of activities with new stimuli, impulsive decision making, and the desire to reward signals (Cloninger et al., 1998). De Fruyt et al. (2000) posited that novelty-seeking is a contrary characteristic to self-determination, and in particular, harm avoidance. In the context of omnichannel, an OCC with high novelty-seeking could be considered a specific type of person who seeks a purchase related to the low dopaminergic activity (Cloninger, 1986). Prior research has shown how in many cases novelty-seeking is a factor that can influence the behavior of consumers (Hirschman, 1980, 1984; Wee et al., 1995; Dabholkar and Bagozzi, 2002; George and George, 2004; Jang and Feng, 2007; Khare et al., 2010; Assaker and Hallak, 2013). Manning et al. (1995) suggested that consumers’ novelty-seeking traits have a positive effect on the adoption of new services. In addition, Rogers and Shoemaker (1971) defined this tendency as the degree of how actively a consumer adopts new things.

From the perspective of omnichannel, consumers’ specific desire to seek new things can be considered as an interaction between the nature of a given service and the need to create unique and frequently varying shopping environments to satisfy this need. Positive experiences with novelty in the context of retail is believed to arouse consumers’ curiosity about the activities of purchasing (Sheth et al., 1991), thus inspiring consumers to carry out purchases (Kim, 2002). Higher levels of enjoyment from novel activities can be associated with consumers’ satisfaction in the context of retail (Anderson and Mittal, 2000), thus inducing consumers to have significantly increased repurchase decisions (Van Birgelen et al., 2006).

Based on prior e-commerce literature, consumers are often shown to be irrational (Gefen et al., 2003). Consumers with high novelty-seeking often focus on something else: for example, in the context of a gamified omnichannel site, OCCs might focus on game mechanics more than the actual value from the purchase. On the other hand, OCCs with low novelty-seeking might focus more on actual value computed on the basis of the probable outcome, as Kahneman and Tversky (2013) claimed in Prospect theory.

Hence, from these perspectives and in the potential context of an omnichannel retailer, we can postulate a hypothesis concerning the causality between novelty-seeking (NS) and repurchase intention (RPI). Accordingly, this research holds that the influence of hedonic value (HV) on repurchase intention (RPI) will increase, as a function of novelty-seeking (NS), while the impact of gamification (GM) on repurchase intention (RPI) and the influence of gamification (GM) on hedonic value (HV) will decrease, as a function of novelty-seeking (NS).

H4: Novelty-seeking influences repurchase intention positively in the context of omnichannel.
H5: Novelty-seeking positively moderates the relation between gamification and hedonic value in the context of omnichannel.
H6: Novelty-seeking negatively moderates the relation between hedonic value and repurchase intention in the context of omnichannel.
H7: Novelty-seeking positively moderates the relation between gamification and repurchase intention in the context of omnichannel.

### Unobserved Heterogeneity

Heterogeneous characteristics such as cultural background (Srite and Karahanna, 2006), demographic characteristics (Venkatesh et al., 2003; Hsieh et al., 2008), and organizational differences (Rai et al., 2006), can often be found as a specific distinction among experimental participant groups (Becker et al., 2013). Comparably, repurchasing behavior of customers can also be distinguished by the perspective of heterogeneity; for instance, carnivore consumers with a higher tendency to risk-take might prefer investing in stock markets than depositing money in banks. Conversely, consumers with a lower tendency to risk-take might prefer to take the opposite action. Heterogeneity in different groups can be quite significant to capture when it can be observed; hence, it is easy for practitioners in the field to refer to the observed heterogeneity in aiding their decision making.

Nevertheless, if there exists unexpected hidden heterogeneity, this unobserved phenomenon can bias the results of experiments and convey inappropriate implications (Ansari et al., 2000; Johns, 2006), which can ensue when the number of samples in the dataset are not sufficient (Becker et al., 2013). Henceforth, unobserved heterogeneity should be considered from the beginning of research because uncovering unobserved heterogeneity can induce acquiring more adequate results as well as provide fruitful findings (Becker et al., 2013).

When it comes to discovering unobserved heterogeneity, however, it is not a simple task, as it is dealing with attributes that are not observable. Thus, it can only be identified with a statistical approach rather than predictions or experience (Hair et al., 2016). Previous studies suggest that various methodologies for discovering unobserved heterogeneity are viable (Becker et al., 2013), i.e., CB-SEM (covariance-based structural equation modeling) (Muthén, 1989; Jedidi et al., 1997; Ansari et al., 2000), conjoint analysis (DeSarbo et al., 1995; Lenk et al., 1996; Gilbride et al., 2006), a panel data model (Allenby and Rossi, 1998; Leszczyc and Bass, 1998), and regression analysis (Späth, 1979; DeSarbo and Cron, 1988; Wedel and DeSarbo, 1994).

Upon reviewing articles published since 2012 (summarized in Table 1), we found that many have addressed gamification within several important domains. Nonetheless, to the best of our knowledge, no study has discussed unobserved heterogeneity alongside the context of gamification; therefore, RQ2 was proposed to fill this gap in the previous literature.

**TABLE 1:** Former research on gamification.

Author(s)	Domain	UH
Eisingerich et al., 2019	Customer engagement	N/A
Hamari and Koivisto, 2015	Gamification service	
Harwood and Garry, 2015	Customer engagement	
Hassan et al., 2019	Motivational feedback	
Helmefalk and Marcusson, 2019	Servicescape context	
Hsu and Chen, 2018	Marketing activities	
Huotari and Hamari, 2012	Service marketing	
Hwang and Choi, 2019	Loyalty programs	
Jang et al., 2018	Mobile exercise application	
Koivisto and Hamari, 2019	Motivational information systems	
Kuo and Chuang, 2016	Online academic dissemination	
Leclercq et al., 2018	Online co-creation communities	
Liu et al., 2017	Information system	
Liu et al., 2018	Smartphone-based job design	
Morford et al., 2014	Behavior analysis and game design	
Moro et al., 2019	Hotel online review’s traits	
Pace and Dipace, 2015	Learning for tourist operators	
Suh and Wagner, 2017	Knowledge contribution	
Suh et al., 2017	Workplace	
Suh et al., 2018	User engagement	
Tobon et al., 2019	Online consumer decision	
Toda et al., 2019	Educational-social networks	
Wu and Seidmann, 2018	Business decision making	
Xi and Hamari, 2019	Intrinsic need satisfaction	
This research	Omnichannel	Yes

*UH, Unobserved Heterogeneity.*

Prior studies on unobserved heterogeneity have adopted its utilization in finding the adequacy of results (Becker et al., 2013), or for classifying data into heterogeneous groups (Sánchez-Prieto et al., 2017). One alternative, suggested by Becker et al. (2013) and Kim et al. (2020) indicates the use of the PLS-POS (partial least squares prediction-oriented segmentation) approach in PLS-SEM. This approach’s merits fall into finding a latent class that can classify the dataset in order to find hidden characteristics (Sarstedt et al., 2019). As OCCs demands and usage needs are unpredictable and varied, investigating unobserved heterogeneity that might exist can help to uncover any variance in users’ responses. Based on this, we propose the following hypothesis:

H8: There exists unobserved heterogeneity in the relationship between hedonic value, gamification, repurchase intention, and novelty-seeking in the context of omnichannel.

## Methodology

### Research Model

**FIGURE 1:**
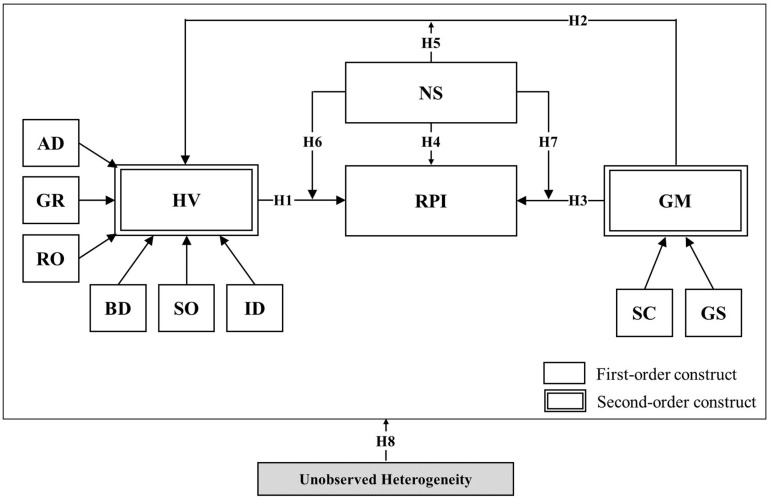
Proposed initial research model in the context of omnichannel retailing.

The following research variables were formulated based on the proposed hypotheses. In the context of omnichannel, we propose that hedonic value (HV) acts as an antecedent of repurchase intention (RPI), gamification (GM) acts as an antecedent of hedonic value (HV), and finally repurchase intention (RPI), alongside novelty-seeking (NS), acts as an antecedent of repurchase intention (RPI). Additionally, we propose that novelty-seeking has a moderating effect on the relationships among hedonic value (HV), gamification (GM), and repurchase intention (RPI). Our proposed research model is depicted in Figure 1.

### Research Data

The dataset for this research was collected through Amazon Mechanical Turk^1^ and was based on 516 participants [female (42.2%) and male (57.8%)] who had purchase experience in using omnichannel retailers. In our survey we provided the definition and example of a three-dimensional omnichannel framework, channel stage, channel type, and channel agent, suggested by Saghiri et al. (2017). Five manipulation questions were inserted into the final survey and acted as a buffer to participants not answering in a correct manner (i.e., “What is this survey about?”), and if the participant answered one of these questions incorrectly, then the survey terminated immediately. The final survey sample included 440 participants out of a total of 516, excluding 76 participants (59 female participants and 17 male participants), who had failed one of the manipulation checks. Participants had diverse omnichannel experience, including shopping on Amazon, eBay, Walmart, and Flipkart. The mean age of the final sample was 31.0 years old, with females (36.1%) and males (63.9%) both represented in the sample. The key demographic descriptive statistics are shown in Table 2.

**TABLE 2:** Key composition of the dataset.

Characteristics		Frequency	%	Cumulative %
Gender	Female	159	36.1	36.1
	Male	281	63.9	100.0
Age	20 to 29	244	55.5	55.5
	30 to 39	143	32.5	88.0
	40 to 49	32	7.3	95.3
	50 and over	21	4.7	100.0
Omnichannel comfortableness	Very comfortable	128	29.1	29.1
	Somewhat comfortable	179	40.7	69.8
	Neither comfortable/uncomfortable	110	25.0	94.8
	Somewhat uncomfortable	18	4.1	98.9
	Very uncomfortable	5	1.1	100.0
Job	Employed for wages	274	62.2	62.2
	Self-employed	132	30.0	92.2
	Out of work	10	2.3	94.5
	A homemaker	10	2.3	96.8
	A student	11	2.5	99.3
	ETC	3	0.7	100.0
Income	Less than $30,000	190	43.2	43.2
	$30,000 ∼ $59,999	147	33.4	76.6
	$60,000 ∼ $89,999	74	16.8	93.4
	$90,000 or more	29	6.6	100.0
Total		440	100.0	100.0

**FIGURE 2:**
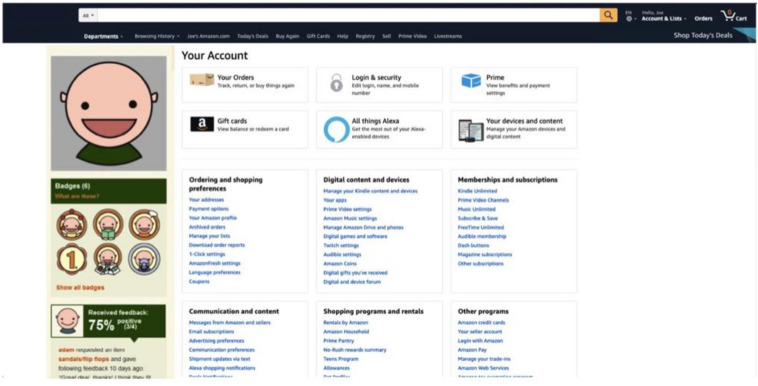
The conceptual omnichannel retailer with gamification components-1.

**FIGURE 3:**
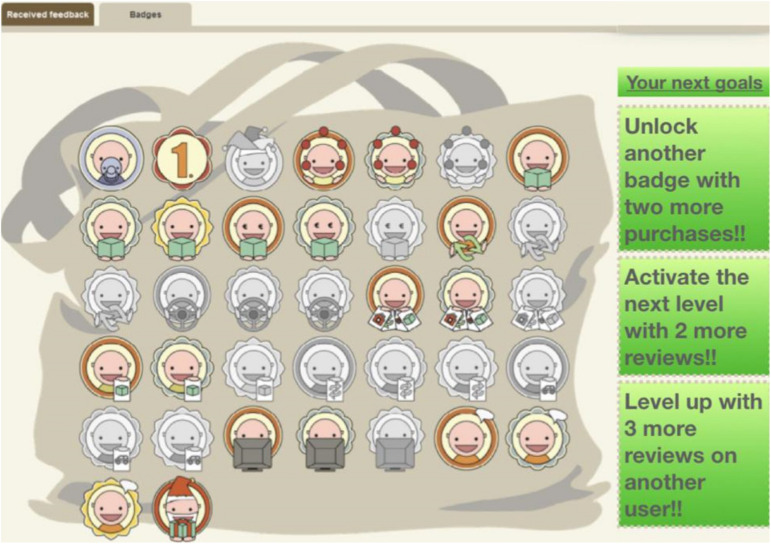
The conceptual omnichannel retailer with gamification components-2.

To manipulate game components on the omnichannel context (e.g., Hamari, 2013), we have introduced participants to a hypothetical scenario describing a virtual shop with game components, as shown in Figures 2, 3. Note that these components were adopted from the prior study of Hamari (2013) and modified using Amazon’s website^2^.

### Measures

The items in the dataset were measured using a seven-point Likert scale that ranged from “strongly disagree (1)” to “strongly agree (7).” The mean and standard deviation values of the constructs, as well as theoretical backgrounds, are presented in Table 3. To validate the consistency of logic, the relevance of context, and ease of understanding, we refined the original version of the questionnaire after conducting a small-scale pretest of the survey questionnaire with 30 respondents with an omnichannel experience. Note that the full questionnaire and the matrix of cross-loadings are presented in Appendices A, B.

**TABLE 3:** Constructs and related literature.

Measurement		Mean	STDEV	Source
Hedonic value (HV)	Adventure (AD)	5.35	1.38	Arnold and Reynolds, 2003
	Gratification (GR)	5.19	1.51	
	Role (RO)	5.51	1.33	
	Best deal (BD)	5.65	1.20	
	Social (SO)	4.94	1.77	
	Idea (ID)	5.30	1.54	
Gamification (GM)	Social comparison (SC)	5.05	1.58	Hamari, 2013
	Goal setting (GS)	5.13	1.57	
Novelty-seeking (NS)		5.47	1.29	Bearden et al., 1989
Repurchase intention (RPI)		5.79	1.18	Parasuraman et al., 2005; Flavián and Guinalíu, 2006

The experiments for this research were conducted using partial least squares (PLS) with SmartPLS 3.0, which has been widely used for experimental purposes to verify hypotheses in a variety of settings including e-commerce (Gefen, 2002; Gefen and Straub, 2004; Kim et al., 2008; Anderson and Swaminathan, 2011; Yoo et al., 2013; Chen and Shen, 2015; Das et al., 2019). PLS is an appropriate approach to reveal whether causalities exist between constructs, shedding light on viability as well as steering supplementary discoveries based on the results (Fornell and Lacker, 1981).

In this paper, we adopted two formative, endogenous, second-order constructs: hedonic value, with six dimensions (adventure, gratification, role, best deal, social, and idea), and gamification, with two dimensions (social comparison and goal setting). For these formative, endogenous, second-order constructs, we used the repeated indicator approach that Chin et al. (2003) suggested. In addition, we used the latent variable scores for the final model (Loch et al., 2003; Marakas et al., 2007; Gaskin and Godfrey, 2014). By adopting this two-stage approach, our model was able to adequately predict second-order formative constructs without any flooding-out effect that repeated indicators can cause (Gaskin and Godfrey, 2014).

The product indicator approach for the moderating effect of the relationship in the PLS-SEM models can only be applied to reflective constructs (Chin et al., 2003). Henceforth, in order to identify the moderating effect of novelty-seeking (NS) on the relationships between hedonic value and repurchase intention, gamification and repurchase intention, and gamification and hedonic value, we have implemented a multi-group analysis (Chin and Dibbern, 2010; Sarstedt et al., 2011). This was applied with the use of the PLS-MGA function embedded within the SmartPLS software.

Furthermore, we strived to reveal the hidden, unobserved heterogeneity in the model by comparing the global model results with unobserved heterogeneity (UH) models by the PLS-POS technique of SmartPLS. PLS-POS is a non-parametric data segmentation technique that needs no prior variable in the analysis of the heterogeneity of scores. Additionally, PLS-POS implements a clustering approach with the deterministic assignment of observations, which has no distributional assumptions (Sánchez-Prieto et al., 2017). Lastly, to deter from a local optimum forming in our results, PLS-POS was repeatedly applied from different starting partitions before an accepted model was reached.

## Results

First, there could be a common-method bias on the dataset because all the variables were self-reported. Therefore, the one-factor analysis was applied to address common method bias (Podsakoff and Organ, 1986). The result of the test revealed that only one factor explained 43.3% of the variance, which is less than 50%. Therefore, the critical influence of common-method bias did not appear in the dataset (Podsakoff and Organ, 1986; Podsakoff et al., 2003, 2012).

Next, due to the complexity of our model, a multi-step approach recommended by Gaskin and Godfrey (2014) was applied: we (1) assessed the first-order constructs, then (2) we analyzed the second-order constructs, before (3) testing the final SEM, and (4) revealing unobserved heterogeneity.

### Assessing the First-Order Constructs

First, we evaluated the reliability and validity of the first-order constructs, as shown in Table 4. Cronbach’s alpha shows the internal consistency reliability of our model, ranging from 0.868 to 0.954 (Fornell and Lacker, 1981). Nevertheless, Cronbach’s alpha validation does not always take into direct consideration the outer loadings in the indicator variables. Therefore, composite reliability should be considered as a more representative measurement. Our composite reliability scores ranged from 0.811 to 0.962. Next, in order to establish the convergent validity of the construct level, we also included the average variance extracted (AVE) (Fornell and Lacker, 1981). The results show that the variance extracted were all higher than 0.5 (Kline et al., 2012). Moreover, the results shown in Table 5 suggest that the bivariate relationships between the first-order constructs on the diagonal were more significant than the values of the other constructs in the model (Chin, 1998).

**TABLE 4:** Reliability and validity of the first-order constructs.

Construct		Cronbach’s α	Composite Reliability	AVE
HV	AD	0.883	0.919	0.740
	GR	0.868	0.919	0.791
	RO	0.889	0.923	0.750
	BD	0.821	0.893	0.736
	SO	0.941	0.962	0.894
	ID	0.881	0.927	0.808
GM	SC	0.925	0.947	0.816
	GS	0.932	0.952	0.831
NS		0.954	0.811	0.888
RPI		0.917	0.865	0.917

**TABLE 5:** Bivariate correlations of the first-order constructs.

Construct		AD	GR	RO	BD	SO	ID	SC	GS	NS	RPI
HV	AD	0.860									
	GR	0.720	0.889								
	RO	0.744	0.698	0.866							
	BD	0.686	0.612	0.696	0.858						
	SO	0.668	0.748	0.584	0.488	0.946					
	ID	0.738	0.772	0.709	0.659	0.764	0.899				
GM	SC	0.653	0.707	0.633	0.543	0.787	0.732	0.913			
	GS	0.632	0.697	0.655	0.564	0.752	0.732	0.603	0.912		
NS		0.690	0.645	0.709	0.675	0.619	0.699	0.663	0.661	0.852	
RPI		0.549	0.381	0.608	0.669	0.205	0.456	0.787	0.380	0.573	0.887

### Assessing the Second-Order Constructs

Following the guidelines of Marakas et al. (2007), we validated the significance of the upward dimensional effects for the second-order formative constructs (HV and GM) using a bootstrap analysis with 1,000 samples. As the results show in Table 6, all *t*-values were significant, and these suggest that all the first-order constructs that are included in our second-order formative constructs are substantial.

**TABLE 6:** Dimension effect for the second-order formative constructs.

Construct		Original β	Mean β	STDEV	*t*-Value	*p*-Value
HV	AD→HV	0.235	0.236	0.006	42.297	0.000
	GR→HV	0.167	0.167	0.006	29.644	0.000
	RO→HV	0.244	0.244	0.007	34.829	0.000
	BD→HV	0.175	0.175	0.006	26.943	0.000
	SO→HV	0.157	0.157	0.007	21.785	0.000
	ID→HV	0.182	0.182	0.006	32.747	0.000
GM	SC→GM	0.506	0.506	0.005	99.134	0.000
	GS→GM	0.516	0.517	0.005	99.968	0.000

**FIGURE 4:**
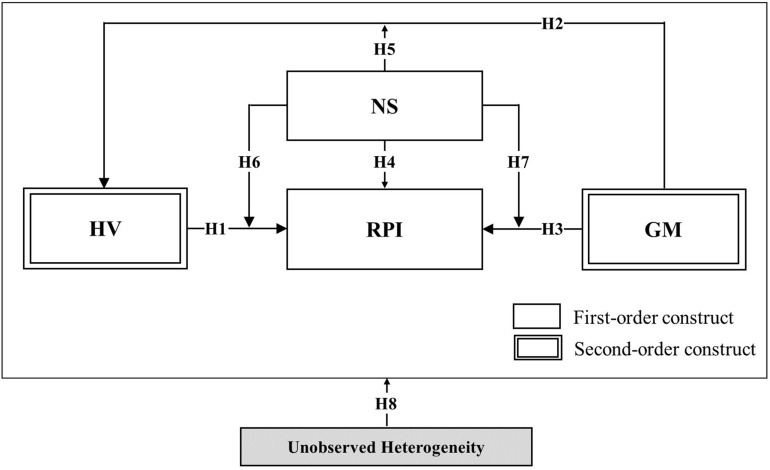
Proposed final research model in the context of omnichannel retailing.

After the validation of the dimension effect for the second-order constructs, based on the guidelines of Marakas et al. (2007), and Loch et al. (2003), latent variable scores for the highest-level constructs were formulated in order to establish the final structural equation model as seen in Figure 4. After the establishment of the final model, we assessed discriminant validity for a pair of two constructs using the heterotrait–monotrait (HTMT) ratio. The liberal threshold values for the HTMT ratio are suggested to be less than 1.00 for discriminant validity (Henseler et al., 2015); see Table 7.

**TABLE 7:**
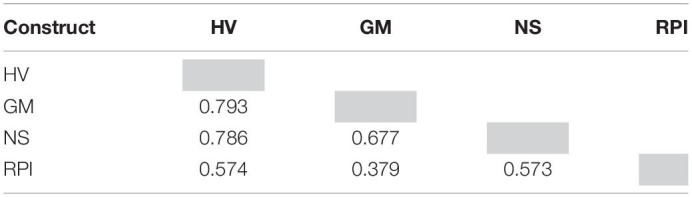
Heterotrait-Monotrait ratio (HTMT) for discriminant validity.

*Gray blank is the standard format of PLS-SEM HTMT.*

### Testing the Global Structural Equation Model

In the context of omnichannel, Hypothesis 1 found that hedonic value influences the repurchase intention of consumers positively (β = 0.499, *p* < 0.001). This indicates that having hedonistic traits such as adventure, gratification, and best deal induces consumers to repurchase. In addition, the result of Hypothesis 2 showed that gamification has a significant positive effect on hedonic value (β = 0.793, *p* < 0.001). These results suggest that gamification has a somewhat strong influence on hedonic value in the context of omnichannel.

**FIGURE 5:**
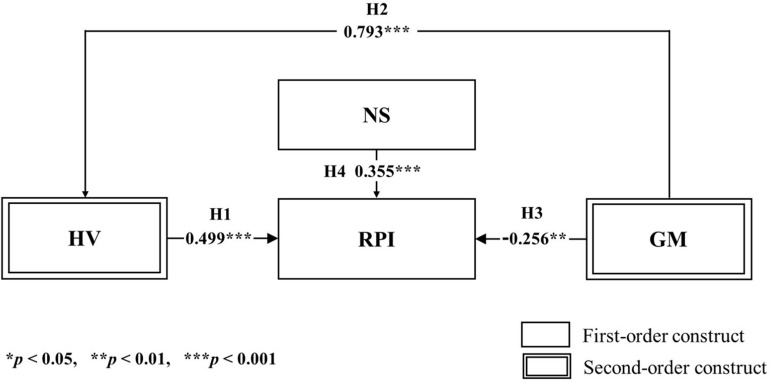
Results of PLS-SEM hypothesis testing on the global model.

For Hypothesis 3, it was discovered that gamification had a significantly negative effect on repurchase intention (β = −0.256, *p* < 0.01), while novelty-seeking had a significantly positive influence on repurchase intention (β = 0.355, *p* < 0.001). Figure 5 below presents the results of our global model analysis.

For the moderating effect of novelty-seeking, Hypotheses 5, 6, and 7 were tested. This part of the analysis represents one of our prime objectives in discovering the moderating effects of consumers’ traits about novelty-seeking, primarily when related to a new feature such as gamification. As Chin et al. (2003) suggest that the product indicator approach should not be applied to formative constructs, we implemented a multi-group analysis (MGA) for our formative second-order constructs (Chiu et al., 2014). The dataset is divided into two novelty-seeking groups, high novelty-seeking (*N* = 208) and low novelty-seeking (*N* = 232), using the median value (Baron and Kenny, 1986) of the sum of three novelty-seeking measurement items.

Where t is the *t*-value with N1 + N2 – 2 degrees of freedom, βk is the path coefficient of the group k, Nk is the sample size of the group k, and SEk is the standard error of the path for group k, the multi-group analysis (MGA) was calculated with the following equation proposed by Keil et al. (2000):

(1) $$t=\frac{\beta_{1}-\beta_{2}}{\sqrt{\frac{{\left(N_{1}-1\right)}^{2}}{N_{1}+N_{2}-2}\cdot SE_{1}^{2}+\frac{{\left(N_{2}-1\right)}^{2}}{N_{1}+N_{2}-2}\cdot SE_{1}^{2}}\times \sqrt{\frac{1}{N_{1}}+\frac{1}{N_{2}}}}$$

Table 8 presents the overall results of the multi-group analysis of our model. For omnichannel consumers with higher novelty-seeking, hedonic value has a smaller influence on their repurchase intention (β = 0.162) than it does on the repurchase intention of consumers with a lower novelty-seeking (β = 0.539). This result supports Hypothesis 6 (*t* = 2.146, *p* < 0.05). On the other hand, gamification has a more considerable influence on repurchase intention for consumers with higher novelty-seeking (β = 0.085) than for consumers with low novelty-seeking (β = −0.344); this supports Hypothesis 7 (*t* = 2.689, *p* < 0.01). However, Hypothesis 5 is not supported. Meaning the difference between consumers with high novelty-seeking and low novelty-seeking was not significant.

**TABLE 8:** Result of multi-group analysis.

Path	*B* (NS High)	β (NS Low)	SE (NS High)	SE (NS Low)	Difference	*t*-Value
GM→HV	0.634	0.739	0.043	0.035	−0.105	1.906
HV→RPI	0.162	0.539	0.142	0.107	−0.377	2.146*
GM→RPI	0.085	−0.344	0.108	0.116	0.429	2.689**

*p < 0.05, **p < 0.01.

### Revealing Unobserved Heterogeneity Models

We initiated the PLS-POS process from a two-group segmentation (*K* = 2). We set the input value of the maximum iteration to 1,000 and the search depth to 440 (our sample size). For an optimization criterion, the sum of target constructs R2 was selected, corresponding to repurchase intention. Lastly, we fixed the stop criterion of finite mixture segmentation at 10, which is necessary to activate the segmenting process. Next, the number of groups (K) was increased in regular sequence by executing the iterative segmentation until one of the subgroup sample sizes became less than the minimum requirement of 10%. Our results showed that with *K* = 3 after two iterative segmentation executions, the smallest group (Group 3, 6.36%) did not meet the requirement, and therefore, “*K* = 2” segmentation was finally selected. From this result, we discovered two subgroups (*K* = 2) from the original dataset. The relative segment sizes of each subgroup are 59.1% (*n* = 260) and 40.9% (*n* = 180). Table 9 shows a summary of the PLS-POS results for the segment retention criteria.

**TABLE 9:** PLS-POS results for segment retention criteria.

Segment (*K*)	Group	HV	RPI	Average *R*^2^	Sizes	%
Original	N/A	0.629	0.393	0.5110	440	100.00%
*K* = 2	Group 1	0.788	0.827	0.6525	260	59.10%
	Group 2	0.522	0.473		180	40.90%
*K* = 3	Group 1	0.594	0.629	0.7110	182	41.36%
	Group 2	0.757	0.730		230	52.27%
	Group 3	0.560	0.996		28	6.36%

**FIGURE 6:**
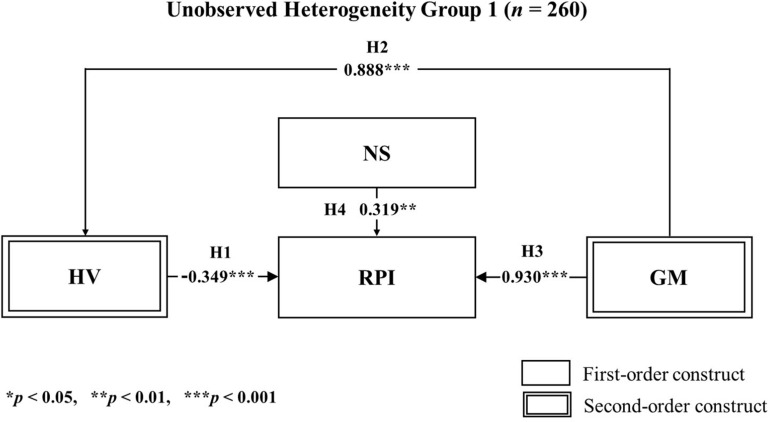
Results of PLS-SEM hypothesis testing on segmented groups according to unobserved heterogeneity group 1 (*n* = 260) Analysis.

**FIGURE 7:**
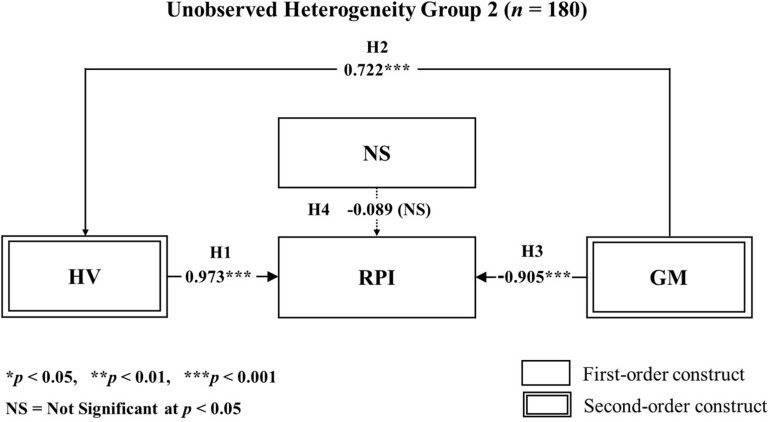
Results of PLS-SEM hypothesis testing on segmented groups according to unobserved heterogeneity group 2 (*n* = 180) Analysis.

After the PLS-POS analysis, we performed an additional PLS-SEM analysis for each subgroup. We discovered that the significance of the hypothetic paths in each unobserved heterogeneity model was statistically different from the paths of the global model. Moreover, the results were reasonably dissimilar between the two unobserved heterogeneity models, as shown in Table 10. Besides, the findings from the unobserved heterogeneity models in the results can be compared clearly with visual representations in Figures 6, 7. Table 11 shows the overall results for our hypothesis testing.

**TABLE 10:** Results of hypothesis tests for segmented groups.

Construct	*B* (Group 1)	*t*-Value (Group 1)	*B* (Group 2)	*t*-Value (Group 2)
HV→RPI	–0.349	3.640***	0.973	9.982***
GM→HV	0.888	43.565***	0.722	18.173***
GM→RPI	0.930	9.357***	–0.905	11.137***
NS→RPI	0.319	3.183**	–0.089	1.008

**p < 0.01, ***p < 0.001.

**TABLE 11:** Overall results of hypotheses tests.

Hypothesis		Results
*H1*	Hedonic value influences repurchase intention positively in the context of omnichannel.	Accepted
*H2*	Gamification influences hedonic value positively in the context of omnichannel.	Accepted
*H3*	Gamification influences repurchase intention positively in the context of omnichannel.	Rejected
*H4*	Novelty-seeking influences repurchase intention positively in the context of omnichannel.	Accepted
*H5*	Novelty-seeking positively moderates the relation between gamification and hedonic value in the context of omnichannel.	Rejected
*H6*	Novelty-seeking negatively moderates the relation between hedonic value and repurchase intention in the context of omnichannel.	Accepted
*H7*	Novelty-seeking positively moderates the relation between gamification and repurchase intention in the context of omnichannel.	Accepted
*H8*	There exists unobserved heterogeneity in the relationship between hedonic value, gamification, repurchase intention, and novelty-seeking in the context of omnichannel.	Accepted

Lastly, it is necessary to control for such variables to exclude any other influential factors. Therefore, five control variables, such as gender, age, omnichannel comfortableness, job, and income, were tested to avoid probable spurious effects. As a result, none of them had a significant influence at *p* < 0.05 for all three research models (global model, unobserved heterogeneity groups 1 and 2).

## Discussion and Implications

In this research, we explored in detail hedonic values infused with gamified features while using novelty-seeking as a moderating effect on repurchase intention within an omnichannel setting. Based on the approach proposed by Gaskin and Godfrey (2014), our analysis found support for a positive influence of hedonic value on repurchase intention (*t* = 0.499^∗∗∗^), when gamification is an antecedent of hedonic value (*t* = 0.793^∗∗∗^). In addition, the result of the moderating effect of novelty-seeking was supported in influencing repurchase intention within our research model (*t* = 0.355^∗∗∗^). Interestingly, gamification itself had a negative effect upon repurchase intention (*t* = −0.256^∗∗^), showing that at face value, without hedonic values, gamification cannot be considered a viable feature within an omnichannel platform. Based on the global structural equation model implemented, we suggest that hedonic value has a strong influence on the repurchase intention of consumers. Conversely, while gamification negatively influenced the repurchase intention of consumers, it had a significantly positive influence on hedonic value. Therefore, we argue that gamification will be a robust technique to induce consumers to repurchase when it is combined with the components of hedonic value (adventure, gratification, role, best deal, social, and idea) in the context of omnichannel. Meanwhile, novelty-seeking, a behavioral characteristic of consumers, was shown to be positively related to consumers’ repeat purchase intention. This result showed that consumers with novelty-seeking characteristics are more likely to purchase again on the same omnichannel website in order to discover a “novel attribute.” Novelty-seeking has been suggested as one of the strong traits in consumers’ behavior related to repurchase intention (Hirschman, 1980; George and George, 2004; Jang and Feng, 2007; Khare et al., 2010; Assaker and Hallak, 2013). Interestingly, our results are counter to those seen previously within the omnichannel literature (Juaneda-Ayensa et al., 2016). We attribute this to the fact that gamification was added into the hedonic value synthesis and thus prior findings may stand true when we are just dealing with hedonic values in isolation.

Next, when we applied an unobserved heterogeneity analysis, we discovered some unique findings within the data. For instance, in the unobserved heterogeneity model 1 (*n* = 208), the initially positive influence of hedonic value on repurchase intention was no more prolonged, with a negative influence (*t* = −0.349^∗∗∗^) prevailing. Also, the initially negative influence of gamification on repurchase intention was reversed into a positive influence (*t* = 0.930^∗∗∗^), suggesting that consumer values when it comes to omnichannel are diverse. By dividing participants into a high novelty-seeking and a low novelty-seeking group, we discovered moderating effects on the paths using the multi-group analysis. Based on the PLS-MGA, we found significant evidence for the moderating effect of novelty-seeking on repurchase intention. This was present when hedonic value and gamification were antecedents of this relationship. As seen in this research, when we focus the attention of the consumers’ behaviors to ones that involve hedonic values, novelty-seeking behavior acts as a vital entity for consumers’ repurchase intention. Previous research has focused on hedonic value as a mostly sybaritic benefit that is related to irrational decisions (Arnold and Reynolds, 2003; To et al., 2007; O’Brien, 2010; Chiu et al., 2014).

As identified by Hamari (2013), gamification is an attempt to convert utilitarian values into hedonic ones, and thus when gamification is present, consumers’ perception of hedonic value might not be very different from values, such as utilitarian ones. Further, the results of our research hint that consumers with high novelty-seeking might be more willing to embrace hedonic value as a compulsory benefit that omnichannel retailers should consider in a more holistic way, including potential benefits a function like gamification can provide to hedonic value. Since the use of omnichannel has become ever more normalized within society, consumers’ tolerance of the dangers of omnichannel shopping is seemingly decreasing. Corporations engaged in omnichannel now must make more of an effort to provide more innovative events, such as gamification, to engage new customers as well as keep their attention on the site in the hope of their making repurchase decisions.

### Implications for Theory

Means-end chain (MEC) theory claims that consumers have three levels of cognitive abstraction based on a hierarchical structure (Gutman, 1982). This paper puts focus on the last two elements of MEC theory, namely, consequences and values. In this hierarchy of goals, Chiu et al. (2014) explain that when it comes to omnichannel, consumers consume a product mostly due to the values gained, and thus values act as the highest serving goals of a consumer (Chiu et al., 2014). Based on this prior research, a major finding in this study was in finding unobserved heterogeneity in consumers and identifying varying values when it comes to the primary constructs within this study. Our research provided an alternative perspective to the one presented through the global model, whereby a dormant, biased interpretation is possible. There are three attributable differences observed between both models. First, the global model identified the significant positive influence of hedonic value on repurchase intention in the context of omnichannel; however, the first unobserved heterogeneity models identified the opposite result. Next, from the perspective of omnichannel, gamification’s significant negative influence on repeat purchase intention became interestingly positive in the first unobserved heterogeneity group. Finally, the trait of consumers’ novelty-seeking, a positive causality with repurchase intention in the context of omnichannel, was insignificant in the second unobserved heterogeneity group, while the positive influence of hedonic value and negative influence of gamification on repurchase intention became much stronger in the second unobserved heterogeneity model compared to that of the global model.

These findings can be conceptualized in the following way. First, unobserved heterogeneity is vital in uncovering unknown differences within consumers repurchase intention; thus, the relationship among hedonic value, gamification, and novelty-seeking in the context of omnichannel were ultimately found within a subset of the respondents. With omnichannel’s fierce competition ever-increasing, omnichannel businesses need to develop appropriate schemes that can be applied to different types of consumers, which are inherently diverse. Based on the discoveries from our unobserved heterogeneity models, we advance a new terminology that segments consumers into two distinct groups to help firms to break through the relentless omnichannel environment: (1) novelty seekers and (2) stand patters.

First, novelty seekers, the first unobserved heterogeneity group, are a highly unique type of consumer. Novelty seekers are not fastidious about evaluating the typical values that omnichannel retailers can provide. Instead, their focus is on new ways or forms of using omnichannel. Furthermore, they are relatively less neurotic about “value” from omnichannel to make their repurchase decisions, compared to traditional consumers. Novelty seekers do not pay a substantial amount of attention to deciding about repurchases according to the value of the product attributes themselves, as long as they can find novelty from the omnichannel site. Therefore, novelty such as gamification could become a panacea for attracting novelty seekers to one’s website. This behavioral characteristic of novelty seekers implies that omnichannel retailers that focus more on innovative products or services should provide enough novel advantages to entice consumers, and gamification might be one of the critical solutions for them.

Next, standpatters, the second unobserved heterogeneity group, are a relatively conservative and common type of consumer. This type of consumer tends to refuse or hesitate to make a repurchase decision with something he/she does not know well. Standpatters rely on prominently typical values such as hedonic value. Additionally, although not directly measured in this paper, we can presume, based on prior research results, they value utilitarian values (Overby and Lee, 2006) for their repurchase decisions. Therefore, unacquainted services, products, functions, and events like gamification could be counterproductive to standpatters because of their robust preference for stability and familiarity. From the viewpoint of a stand-patter, emphasizing the use of unnecessary gamified functions rather than the fundamental value of products or services on a platform could be harmful. Hence, in order to prevent standpatters from leaving, omnichannel retailers need to make sure gamified options are presented as opt-ins, as opposed to being presented forcefully.

### Practical Implications

For omnichannel sellers engaged in the current, fierce omnichannel market, this study has the following implications. First, it sheds light on the potential adoption of gamification within an omnichannel setting. To the best of our knowledge, this is the first paper to attempt this, and thus for practitioners involved in omnichannel, the results from this study give essential information for moving forward with the potential adoption of gamification. Based on the results, it implies that hedonic value is still crucial to satisfying omnichannel customers’ needs. However, as shown in our model, the implementation of gamification alongside hedonic values is a viable construct for customers who inherently have novelty-seeking behaviors. Thus, the application of badges or levels within the customer experience, i.e., the inclusion of badges for doing specific tasks such as leaving reviews or buying a product in bulk, may induce people to increase their repeat purchasing while having fun. Within this paper, we do not suggest an optimum gamification solution; however, we bring to light the potential adoption of gamification. For a full review of the many gamification functions, see Hamari and Koivisto (2015).

Next, the unobserved heterogeneity showed that some consumers were in favor of gamification, and some were not. As a marketing strategy, including website design, gamification should be considered something people should be able to opt in to freely. Forcing consumers who do not show high novelty-seeking traits and prefer more traditional values such as utilitarian and hedonic value (Overby and Lee, 2006) to engage in gamification may cause them to leave the website. Thus, we recommend that the inception of gamification should be done in beta stages to find out the reaction of certain small groups of users at first. In addition to the above implications, marketers implementing omnichannel also need to pay great attention to the demographic phenomena shown in the results of hidden heterogeneity analyses. Considering that the first unobserved heterogeneity group accounted for 59.1% of the total (260 out of 440 respondents), novelty seekers might not be an exclusive type of consumer anymore. Instead, this provocative kind of consumer, who is continuously looking for something sensational, might become a new normal in the context of omnichannel. In contrast, a traditional and common type of consumers in the second unobserved heterogeneity group (40.9%) might not be a standard kind to any further extent in the rapidly transforming retailing market. Sometimes, things that have been considered insignificant, such as gamification, may in fact be more valuable if we look at it in detail. Due to the evolved information technology available to most consumers, access to various information has never been so easy. For this reason, it is clear that consumers are less likely to open their wallets to the traditional sales strategies of firms. Henceforth, marketing practitioners in the omnichannel retailing industry should reserve the extra capacity to take account of pertinent consumer traits that might be shrouded in the heterogeneity like the phenomenon from this study. In other words, they might need to consider and deliver more intriguing concepts such as gamification to satisfy these novelty-seeking omnichannel consumers. This is also a pertinent point which could also be relevant to marketers and practitioners working under different yet similar retail systems.

### Limitations

Firstly, this study only implemented a cross-sectional analysis of the target problem. Specifically, we performed a cross-sectional survey, which is potentially susceptible to standard method variance (Podsakoff et al., 2012). To eliminate such bias, a longitudinal study needs to be considered in order to validate if any causation exists between the variables. Of course, with panel data or longitudinal data, this study’s findings could have potentially higher generalizability as well as more of an indication of true causation for all the variables. Thus, we admit that this study acts more as an observation of the potential implication of gamification within the omnichannel context than a concrete concept of gamification within omnichannel. Of course, this could be eradicated through future methodologies that allow for a more skillful manipulation of variables in models (Antonakis et al., 2010).

The next limitation is with the sampling methodology and manipulation. Due to the current world circumstance whereby most shops are shut due to COVID-19, it was not feasible to undertake the original plan for the study whereby we wanted to undertake the survey based on a sampling technique whereby participants were selected based on physical proximity to an omnichannel store. Of course, this is a future research agenda that will be discussed in the next section. For this reason, we cannot guarantee that we were able to capture an omnichannel scenario compared to for example an e-commerce or multichannel scenario. Although we attempted to prime the participants into thinking about an omnichannel context and placed manipulation checks within the survey, there is a chance that some participants answered with another retail system in mind. Additionally, the young age of the survey participants was a limitation. This unbalanced density of age groups was due to younger groups’ specific characteristics of seeking novelty in the gamified components (Bittner and Shipper, 2014; Koivisto and Hamari, 2014); thus, the survey patrons who had significant interest in mTurk might be relatively young. Another limitation with the study can be seen in our failure to capture channel integration. However, we argue that gamification is not unique to one channel and could be easily integrated into any omnichannel retail infrastructure.

Next, as the results from unobserved heterogeneity (model one versus model two) fluctuated at quite a significant rate in terms of consumers’ values for repurchase intention, the results from the analysis suggest it might have a higher potential for the Type I error that Becker et al. (2013) proposed. Therefore, we recommend this research begins as a starting ground for other research and hope that future research can verify whether this research has, in fact, a Type I error or not. This will help clarify whether or not consumers do have such varying behaviors.

### Future Research Issues

Firstly, future research can investigate demographical differences. Prior research on gamification has shown that women report enormous social benefits of using gamification in persuasive technology (Koivisto and Hamari, 2014). We suggest that demographics, including ethnicity, i.e., Western versus Asian consumers, could be included in future research on gamification within omnichannel. Further, we did not analyze any differences in products when it comes to omnichannel. However, certain products, i.e., everyday products, are products that need to be purchased regularly, and thus gamification might be more suitable for these types of purchases. However, more exceptional points or levels could be awarded for bigger-ticket items; therefore, trying to capture and understand the dynamics that could be in play may serve as a good future research direction.

Another future recommendation is in the sampling technique. Obtaining participants we could be sure had experience in an omnichannel setting would only be feasible by recruiting from omnichannel stores. Preferably, these would be participants who were visiting offline stores after having been actively engaged in other parts of the omnichannel system. In addition, creating an offline version of the gamified concept would help to give a more hands-on experience, i.e., in-store events that would have some of the gamified components included. This could be a gamified points system for a specific purchase as one example.

## Data Availability Statement

The datasets presented in this article are not readily available because the datasets cannot be shared without participant’s prior consent. Requests to access the datasets should be directed to KL, kunchanglee@gmail.com.

## Ethics Statement

The studies involving human participants were reviewed and approved by IRB of Sungkyunkwan University. The patients/participants provided their written informed consent to participate in this study.

## Author Contributions

CK designed the experiment, collected and analyzed the data, and drafted and revised the manuscript. FC assisted with the experiment, analyzed the data, and drafted and revised the manuscript. KL supervised the experimental design and the data collection and revised the manuscript. All authors contributed to the article and approved the submitted version.

## Conflict of Interest

CK was employed by company Airports Council International (ACI) World. The remaining authors declare that the research was conducted in the absence of any commercial or financial relationships that could be construed as a potential conflict of interest.

## Footnotes

1 https://www.mturk.com

2 https://www.amazon.com

## Appendix

**TABLE A:** Items for questionnaire.

Construct	Measurement Items	
**Hedonic value (HV)**		
Adventure (AD)	AD1	To me, shopping on this omnichannel retailer is an adventure.
	AD2	I find shopping on this omnichannel retailer stimulating.
	AD3	Shopping on this omnichannel retailer is a thrill for me.
	AD4	Shopping on this omnichannel retailer makes me feel like I am in my own universe.
Gratification (GR)	GR1	When I am in a low mood, I go shopping in this omnichannel retailer to make me feel better.
	GR2	To me, shopping on this omnichannel retailer is a way of relieving stress.
	GR3	I go shopping in this omnichannel retailer when I want to treat myself to something special.
Role (RO)	RO1	I like shopping in this omnichannel retailer for others because, when they feel good, I feel good.
	RO2	I feel good when I buy things on this omnichannel retailer for the special people in my life.
	RO3	I enjoy shopping in this omnichannel retailer for my friends and family.
	RO4	I enjoy shopping around this omnichannel retailer to find the perfect gift for someone.
Best deal (BD)	BD1	For the most part, I go shopping in this omnichannel retailer when there are sales.
	BD2	I enjoy looking for discounts when I shop in this omnichannel retailer.
	BD3	I enjoy hunting for bargains when I shop on this omnichannel retailer.
Social (SO)	SO1	I go shopping in this omnichannel retailer with my friends and family in order to socialize.
	SO2	I enjoy socializing with others when I shop in this omnichannel retailer.
	SO3	Shopping on this omnichannel retailer with others is a bonding experience.
Idea (ID)	ID1	I go shopping in this omnichannel retailer to keep up with the trends.
	ID2	I go shopping in this omnichannel retailer to keep up with new fashions.
	ID3	I go shopping in this omnichannel retailer to see what new products are available.
**Gamification (GM)**		
Social comparison (SC)	SC1	In order to unlock access to (and/or upgrade) a badge (avatar) and be able to compare with other users, I would browse a site’s items more.
	SC2	In order to unlock access to (and/or upgrade) a badge (avatar) and be able to compare with other users, I would purchase more items.
	SC3	Having access to and being able to compare badges (avatars) with other users will likely increase me posting comments (i.e., reviews/recommendations).
	SC4	Having access to and being able to compare badges (avatars) with other users will increase the number of different pages I view (i.e., look at other users’ pages).
Goal setting (GS)	GS1	If I am presented with clear goals on how to access or obtain badges (avatars), I will browse a site’s items more.
	GS2	If I am presented with clear goals on how to access or obtain badges (avatars), I will purchase more items.
	GS3	If I am presented with clear goals on how to access or obtain badges (avatars), I will post more comments (i.e., reviews/recommendations).
	GS4	If I am presented with clear goals on how to access or obtain badges (avatars), I will increase the number of different pages I view (i.e., look at other users’ pages).
Novelty-seeking (NS)	NS1	I tend to pursue new ideas or experiences.
	NS2	I like to experience new things and make changes in my daily life.
	NS3	I am more interested in using new products or services than other people.
Repurchase intention (RPI)	RPI1	I plan to continue using this omnichannel retailer to purchase products.
	RPI2	I consider this omnichannel retailer to be my first choice for transactions in the future.
	RPI3	It is likely that I will continue purchasing products from this omnichannel retailer in the future.

**TABLE B:** Cross-loadings.

	AD	GR	RO	BD	SO	ID	SC	GS	NS	RPI
AD1	**0.861**	0.630	0.627	0.628	0.602	0.653	0.558	0.520	0.605	0.465
AD2	**0.844**	0.576	0.644	0.610	0.476	0.589	0.491	0.487	0.580	0.500
AD3	**0.879**	0.631	0.642	0.589	0.545	0.606	0.541	0.522	0.607	0.490
AD4	**0.858**	0.640	0.649	0.534	0.670	0.690	0.654	0.641	0.584	0.438
GR1	0.631	**0.907**	0.583	0.541	0.743	0.704	0.679	0.648	0.571	0.299
GR2	0.622	**0.888**	0.575	0.488	0.692	0.676	0.627	0.639	0.550	0.274
GR3	0.668	**0.872**	0.700	0.601	0.565	0.679	0.581	0.573	0.599	0.440
RO1	0.679	0.726	**0.858**	0.556	0.636	0.711	0.659	0.640	0.637	0.433
RO2	0.610	0.579	**0.861**	0.556	0.479	0.590	0.496	0.530	0.589	0.512
RO3	0.667	0.582	**0.894**	0.649	0.484	0.591	0.546	0.562	0.646	0.575
RO4	0.617	0.518	**0.852**	0.654	0.410	0.553	0.481	0.530	0.580	0.597
BD1	0.622	0.578	0.591	**0.851**	0.500	0.635	0.536	0.543	0.608	0.534
BD2	0.553	0.493	0.600	**0.866**	0.362	0.542	0.427	0.452	0.557	0.578
BD3	0.586	0.499	0.600	**0.857**	0.387	0.513	0.428	0.450	0.571	0.613
SO1	0.605	0.692	0.521	0.449	**0.945**	0.709	0.744	0.702	0.563	0.160
SO2	0.631	0.721	0.560	0.448	**0.940**	0.727	0.738	0.713	0.585	0.188
SO3	0.658	0.710	0.575	0.487	**0.952**	0.732	0.750	0.719	0.606	0.233
ID1	0.675	0.741	0.633	0.563	0.776	**0.927**	0.711	0.697	0.634	0.339
ID2	0.671	0.715	0.610	0.547	0.757	**0.914**	0.706	0.701	0.603	0.331
ID3	0.646	0.623	0.670	0.671	0.522	**0.856**	0.553	0.573	0.648	0.567
SC1	0.586	0.624	0.578	0.493	0.681	0.654	**0.903**	0.797	0.624	0.354
SC2	0.583	0.648	0.519	0.478	0.729	0.677	**0.884**	0.816	0.571	0.276
SC3	0.596	0.642	0.585	0.478	0.729	0.662	**0.918**	0.850	0.597	0.329
SC4	0.596	0.641	0.605	0.512	0.704	0.654	**0.908**	0.835	0.605	0.349
GS1	0.558	0.631	0.610	0.525	0.680	0.664	0.827	**0.915**	0.615	0.394
GS2	0.583	0.639	0.548	0.490	0.731	0.677	0.815	**0.897**	0.602	0.278
GS3	0.580	0.645	0.618	0.510	0.665	0.665	0.848	**0.919**	0.597	0.356
GS4	0.582	0.625	0.612	0.531	0.668	0.662	0.840	**0.914**	0.597	0.355
NS1	0.589	0.536	0.615	0.622	0.505	0.563	0.573	0.545	**0.866**	0.523
NS2	0.572	0.501	0.588	0.558	0.478	0.561	0.521	0.529	**0.866**	0.492
NS3	0.606	0.622	0.611	0.542	0.610	0.672	0.607	0.624	**0.823**	0.445
RPI1	0.464	0.326	0.538	0.605	0.146	0.396	0.292	0.308	0.525	**0.894**
RPI2	0.516	0.368	0.538	0.580	0.237	0.420	0.358	0.379	0.498	**0.862**
RPI3	0.483	0.320	0.544	0.594	0.165	0.396	0.314	0.324	0.501	**0.905**

*Bold values represent the loadings that are above the recommended 0.5.*

## References

[B1] Allenby G. M. Rossi P. E. (1998). Marketing models of consumer heterogeneity. *J. Econom.* 89 57–78. 10.1016/s0304-4076(98)00055-4

[B2] Anderson E. W. Mittal V. (2000). Strengthening the satisfaction-profit chain. *J. Serv. Res.* 3 107–120. 10.1177/109467050032001

[B3] Anderson R. E. Swaminathan S. (2011). Customer satisfaction and loyalty in e-markets: a PLS path modeling approach. *J. Mark. Theory Pract.* 19 221–234. 10.2753/mtp1069-6679190207

[B4] Ansari A. Jedidi K. Jagpal S. (2000). A hierarchical Bayesian methodology for treating heterogeneity in structural equation models. *Mark. Sci.* 19 328–347. 10.1287/mksc.19.4.328.11789 19642375

[B5] Antonakis J. Bendahan S. Jacquart P. Lalive R. (2010). On making causal claims: a review and recommendations. *Leadersh. Q.* 21 1086–1120. 10.1016/j.leaqua.2010.10.010

[B6] Arnold M. J. Reynolds K. E. (2003). Hedonic shopping motivations. *J. Retail.* 79 77–95. 10.1016/s0022-4359(03)00007-1

[B7] Assaker G. Hallak R. (2013). Moderating effects of tourists’ novelty-seeking tendencies on destination image, visitor satisfaction, and short-and long-term revisit intentions. *J. Travel Res.* 52 600–613. 10.1177/0047287513478497

[B8] Baron R. M. Kenny D. A. (1986). The moderator–mediator variable distinction in social psychological research: conceptual, strategic, and statistical considerations. *J. Pers. Soc. Psychol.* 51 1173–1182. 10.1037/0022-3514.51.6.1173 3806354

[B9] Bearden W. O. Netemeyer R. G. Teel J. E. (1989). Measurement of consumer susceptibility to interpersonal influence. *J. Consum. Res.* 15 473–481.

[B10] Beck N. Rygl D. (2015). Categorization of multiple channel retailing in Multi-, Cross-, and Omni-Channel Retailing for retailers and retailing. *J. Retail. Consum. Serv.* 27 170–178. 10.1016/j.jretconser.2015.08.001

[B11] Becker J.-M. Rai A. Ringle C. M. Völckner F. (2013). Discovering unobserved heterogeneity in structural equation models to avert validity threats. *MIS Q.* 37 665–694. 10.25300/misq/2013/37.3.01

[B12] Bell D. R. Gallino S. Moreno A. (2014). How to win in an omnichannel world. *MIT Sloan Manage. Rev.* 56:45.

[B13] Bittner J. V. Shipper J. (2014). Motivational effects and age differences of gamification in product advertising. *J. Consum. Mark*. 31/5 391–400. 10.1108/jcm-04-2014-0945

[B14] Brown S. A. Venkatesh V. (2005). Model of adoption of technology in households: a baseline model test and extension incorporating household life cycle. *MIS Q.* 399–426.

[B15] Brynjolfsson E. Hu Y. J. Rahman M. S. (2013). *Competing in the Age of Omnichannel Retailing.* Cambridge, MA: MIT.

[B16] Chen J. Shen X.-L. (2015). Consumers’ decisions in social commerce context: an empirical investigation. *Decis. Support Syst.* 79 55–64. 10.1016/j.dss.2015.07.012

[B17] Chin W. W. (1998). *The Partial Least Squares Approach to Structural Equation Modeling.* Mahwah, NJ: Lawrence Erlbaum.

[B18] Chin W. W. Dibbern J. (2010). *A Permutation Based Procedure for Multi-Group PLS Analysis: Results of Tests of Differences on Simulated Data and a Cross Cultural Analysis of the Sourcing of Information System Services between Germany and the USA.* Heidelberg: Springer.

[B19] Chin W. W. Marcolin B. L. Newsted P. R. (2003). A partial least squares latent variable modeling approach for measuring interaction effects: results from a Monte Carlo simulation study and an electronic-mail emotion/adoption study. *Inf. Syst. Res.* 14 189–217. 10.1287/isre.14.2.189.16018 19642375

[B20] Chiu C. M. Wang E. T. Fang Y. H. Huang H. Y. (2014). Understanding customers’ repeat purchase intentions in B2C e-commerce: the roles of utilitarian value, hedonic value and perceived risk. *Inf. Syst. J.* 24 85–114. 10.1111/j.1365-2575.2012.00407.x

[B21] Cloninger C. R. (1986). A unified biosocial theory of personality and its role in the development of anxiety states. *Psychiatr. Dev.* 3 167–226. 3809156

[B22] Cloninger C. R. Svrakic D. M. Przybeck T. R. (1998). A psychobiological model of temperament and character. *Dev. Psychiatry Complex.* 50 1–16. 10.1001/archpsyc.1993.01820240059008 8250684

[B23] Cook G. (2014). Customer experience in the omni-channel world and the challenges and opportunities this presents. *J. Direct Data Digit. Mark. Pract.* 15 262–266. 10.1057/dddmp.2014.16

[B24] Dabholkar P. A. Bagozzi R. P. (2002). An attitudinal model of technology-based self-service: moderating effects of consumer traits and situational factors. *J. Acad. Mark. Sci.* 30 184–201. 10.1177/00970302030003001

[B25] Das S. Mishra A. Cyr D. (2019). Opportunity gone in a flash: measurement of e-commerce service failure and justice with recovery as a source of e-loyalty. *Decis. Support Syst.* 125:113130 10.1016/j.dss.2019.113130

[B26] De Fruyt F. Van De Wiele L. Van Heeringen C. (2000). Cloninger’s psychobiological model of temperament and character and the five-factor model of personality. *Pers. Individ. Differ.* 29 441–452. 10.1016/s0191-8869(99)00204-4

[B27] Dedeoglu B. B. Bilgihan A. Ye B. H. Buonincontri P. Okumus F. (2018). The impact of servicescape on hedonic value and behavioral intentions: the importance of previous experience. *Int. J. Hosp. Manage.* 72 10–20. 10.1016/j.ijhm.2017.12.007

[B28] DeSarbo W. S. Cron W. L. (1988). A maximum likelihood methodology for clusterwise linear regression. *J. Classif.* 5 249–282. 10.1007/bf01897167

[B29] DeSarbo W. S. Ramaswamy V. Cohen S. H. (1995). Market segmentation with choice-based conjoint analysis. *Mark. Lett.* 6 137–147. 10.1007/bf00994929

[B30] Eisingerich A. B. Marchand A. Fritze M. P. Dong L. (2019). Hook vs. hope: how to enhance customer engagement through gamification. *Int. J. Res. Mark.* 36 200–215. 10.1016/j.ijresmar.2019.02.003

[B31] Escobar-Rodríguez T. Carvajal-Trujillo E. (2014). Online purchasing tickets for low cost carriers: an application of the unified theory of acceptance and use of technology (UTAUT) model. *Tourism Manage.* 43 70–88. 10.1016/j.tourman.2014.01.017

[B32] Fang Y.-H. Chiu C.-M. Wang E. T. (2011). Understanding customers’ satisfaction and repurchase intentions: an integration of IS success model, trust, and justice. *Internet Res.* 21 479–503. 10.1108/10662241111158335

[B33] Fassnacht M. Koese I. (2006). Quality of electronic services: conceptualizing and testing a hierarchical model. *J. Serv. Res.* 9 19–37. 10.1177/1094670506289531

[B34] Festinger L. (1954). A theory of social comparison processes. *Hum. Relat.* 7 117–140. 10.1177/001872675400700202

[B35] Flavián C. Guinalíu M. (2006). Consumer trust, perceived security and privacy policy: three basic elements of loyalty to a web site. *Ind. Manage. Data Syst.* 106 601–620. 10.1108/02635570610666403

[B36] Fornell C. R. Lacker D. F. (1981). Two structural equation models with unobservable variables and measurement error. *J. Mark. Res.* 18 39–50. 10.1177/002224378101800104

[B37] Fortes Tondello G. Premsukh H. Nacke L. (2018). *A Theory of Gamification Principles through Goal-Setting Theory.* Available online at: http://hdl.handle.net/10125/50027 (accessed March 3, 2020).

[B38] Gardner J. W. (1939). Level of aspiration in response to a prearranged sequence of scores. *J. Exp. Psychol.* 25 601–621. 10.1037/h0056070

[B39] Gaskin J. Godfrey S. (2014). “Successful system-use: It’s not just who you are, but what you do,” in *Proceedings of Special Interest Group on Human Computer Interaction 2014*, Auckland.

[B40] Gefen D. (2002). Customer loyalty in e-commerce. *J. Assoc. Inf. Syst.* 3 27–51.

[B41] Gefen D. Karahanna E. Straub D. W. (2003). Inexperience and experience with online stores: the importance of TAM and trust. *IEEE Trans. Eng. Manage.* 50 307–321. 10.1109/tem.2003.817277

[B42] Gefen D. Straub D. W. (2004). Consumer trust in B2C e-Commerce and the importance of social presence: experiments in e-Products and e-Services. *Omega* 32 407–424. 10.1016/j.omega.2004.01.006

[B43] George B. P. George B. P. (2004). Past visits and the intention to revisit a destination: place attachment as the mediator and novelty seeking as the moderator. *J. Tourism Stud.* 15 51–66.

[B44] Gilbride T. J. Allenby G. M. Brazell J. D. (2006). Models for heterogeneous variable selection. *J. Mark. Res.* 43 420–430.

[B45] Gu J. Z. Tayi G. K. (2017). Consumer pseudo-showrooming and omni-channel product placement strategies. *Manage. Inf. Syst. Q.* 41, 583–606.

[B46] Gutman J. (1982). A means-end chain model based on consumer categorization processes. *J. Mark.* 46 60–72. 10.1177/002224298204600207

[B47] Hair J. Joe F. Sarstedt M. Matthews L. M. Ringle C. M. (2016). Identifying and treating unobserved heterogeneity with FIMIX-PLS: part I–method. *Eur. Bus. Rev.* 28 63–76. 10.1108/ebr-09-2015-0094

[B48] Hamari J. (2013). Transforming homo economicus into homo ludens: a field experiment on gamification in a utilitarian peer-to-peer trading service. *Electron. Commer. Res. Appl.* 12 236–245. 10.1016/j.elerap.2013.01.004

[B49] Hamari J. Koivisto J. (2015). Why do people use gamification services? *Int. J. Inf. Manage.* 35 419–431. 10.1016/j.ijinfomgt.2015.04.006

[B50] Hamari J. Koivisto J. Sarsa H. (2014). “Does Gamification work?-A literature review of empirical studies on Gamification,” in *Proceedings of the 47th Hawaii International Conference on System Sciences HICSS*, Waikoloa, HI, 3025–3034.

[B51] Harwood T. Garry T. (2015). An investigation into gamification as a customer engagement experience environment. *J. Serv. Mark.* 29 533–546. 10.1108/jsm-01-2015-0045

[B52] Hassan L. Dias A. Hamari J. (2019). How motivational feedback increases user’s benefits and continued use: a study on gamification, quantified-self and social networking. *Int. J. Inf. Manage.* 46 151–162. 10.1016/j.ijinfomgt.2018.12.004

[B53] Helmefalk M. Marcusson L. (2019). Gamification in a servicescape context: a conceptual framework. *Int. J. Internet Mark. Advert.* 13 22–46.

[B54] Henseler J. Ringle C. M. Sarstedt M. (2015). A new criterion for assessing discriminant validity in variance-based structural equation modeling. *J. Acad. Mark. Sci.* 43 115–135. 10.1007/s11747-014-0403-8

[B55] Herhausen D. Binder J. Schoegel M. Herrmann A. (2015). Integrating bricks with clicks: retailer-level and channel-level outcomes of online–offline channel integration. *J. Retail.* 91 309–325. 10.1016/j.jretai.2014.12.009

[B56] Hirschman E. C. (1980). Innovativeness, novelty seeking, and consumer creativity. *J. Consum. Res.* 7 283–295.

[B57] Hirschman E. C. (1984). Experience seeking: a subjectivist perspective of consumption. *J. Bus. Res.* 12 115–136. 10.1016/0148-2963(84)90042-0

[B58] Hsieh J. P.-A. Rai A. Keil M. (2008). Understanding digital inequality: comparing continued use behavioral models of the socio-economically advantaged and disadvantaged. *MIS Q.* 97–126.

[B59] Hsu C.-L. Chen M.-C. (2018). How gamification marketing activities motivate desirable consumer behaviors: focusing on the role of brand love. *Comput. Hum. Behav.* 88 121–133. 10.1016/j.chb.2018.06.037

[B60] Huotari K. Hamari J. (2012). “Defining gamification: a service marketing perspective,” in *Proceeding of the 16th International Academic MindTrek Conference* (New York, NY: ACM), 17–22.

[B61] Hwang J. Choi L. (2019). Having fun while receiving rewards?: exploration of gamification in loyalty programs for consumer loyalty. *J. Bus. Res.* 106 365–376. 10.1016/j.jbusres.2019.01.031

[B62] Jang S. Kitchen P. J. Kim J. (2018). The effects of gamified customer benefits and characteristics on behavioral engagement and purchase: evidence from mobile exercise application uses. *J. Bus. Res.* 92 250–259. 10.1016/j.jbusres.2018.07.056

[B63] Jang S. S. Feng R. (2007). Temporal destination revisit intention: the effects of novelty seeking and satisfaction. *Tourism Manage.* 28 580–590. 10.1016/j.tourman.2006.04.024

[B64] Jedidi K. Jagpal H. S. Desarbo W. S. (1997). Finite-mixture structural equation models for response-based segmentation and unobserved heterogeneity. *Mark. Sci.* 16 39–59. 10.1287/mksc.16.1.39 19642375

[B65] Johns G. (2006). The essential impact of context on organizational behavior. *Acad. Manage. Rev.* 31 386–408. 10.5465/amr.2006.20208687

[B66] Juaneda-Ayensa E. Mosquera A. Sierra Murillo Y. (2016). Omnichannel customer behavior: key drivers of technology acceptance and use and their effects on purchase intention. *Front. Psychol.* 7:1117. 10.3389/fpsyg.2016.01117 27516749 PMC4963459

[B67] Kahneman D. Tversky A. (2013). “Prospect theory: an analysis of decision under risk,” in *Handbook of the Fundamentals of Financial Decision Making: Part I* eds MacLean L. C. Ziemba W. T. (Singapore: World Scientific), 99–127. 10.1142/9789814417358_0006

[B68] Keil M. Tan B. C. Wei K.-K. Saarinen T. Tuunainen V. Wassenaar A. (2000). A cross-cultural study on escalation of commitment behavior in software projects. *MIS Q.* 299–325.

[B69] Khare A. Singh S. Khare A. (2010). Innovativeness/novelty-seeking behavior as determinants of online shopping behavior among Indian youth. *J. Internet Commer.* 9 164–185. 10.1080/15332861.2010.529054

[B70] Kim C. Jeon H. G. Lee K. C. (2020). Discovering the role of emotional and rational appeals and hidden heterogeneity of consumers in advertising copies for sustainable marketing. *Sustainability* 12:5189 10.3390/su12125189

[B71] Kim D. J. Ferrin D. L. Rao H. R. (2008). A trust-based consumer decision-making model in electronic commerce: the role of trust, perceived risk, and their antecedents. *Decis. Support Syst.* 44 544–564. 10.1016/j.dss.2007.07.001

[B72] Kim J.-C. Chun S.-H. (2018). Cannibalization and competition effects on a manufacturer’s retail channel strategies: implications on an omni-channel business model. *Decis. Support Syst.* 109 5–14. 10.1016/j.dss.2018.01.007

[B73] Kim Y.-K. (2002). Consumer value: an application to mall and Internet shopping. *Int. J. Retail Distrib. Manage.* 30 595–602. 10.1108/09590550210453075

[B74] King R. C. Schilhavy R. A. Chowa C. Chin W. W. (2016). Do customers identify with our website? The effects of website identification on repeat purchase intention. *Int. J. Electron. Commer.* 20 319–354. 10.1080/10864415.2016.1121762

[B75] Kline E. Wilson C. Ereshefsky S. Tsuji T. Schiffman J. Pitts S. (2012). Convergent and discriminant validity of attenuated psychosis screening tools. *Schizophr. Res.* 134 49–53. 10.1016/j.schres.2011.10.001 22036199

[B76] Koivisto J. Hamari J. (2014). Demographic differences in perceived benefits from gamification. *Comput. Hum. Behav.* 35 179–188. 10.1016/j.chb.2014.03.007

[B77] Koivisto J. Hamari J. (2019). The rise of motivational information systems: a review of gamification research. *Int. J. Inf. Manage.* 45 191–210. 10.1016/j.ijinfomgt.2018.10.013

[B78] Kuo M.-S. Chuang T.-Y. (2016). How gamification motivates visits and engagement for online academic dissemination–An empirical study. *Comput. Hum. Behav.* 55 16–27. 10.1016/j.chb.2015.08.025

[B79] Landers R. N. Bauer K. N. Callan R. C. (2017). Gamification of task performance with leaderboards: a goal setting experiment. *Comput. Hum. Behav.* 71 508–515. 10.1016/j.chb.2015.08.008

[B80] Leclercq T. Hammedi W. Poncin I. (2018). The boundaries of gamification for engaging customers: effects of losing a contest in online co-creation communities. *J. Interact. Mark.* 44 82–101. 10.1016/j.intmar.2018.04.004

[B81] Lemon K. N. Verhoef P. C. (2016). Understanding customer experience throughout the customer journey. *J. Mark.* 80 69–96. 10.1509/jm.15.0420 11670861

[B82] Lenk P. J. Desarbo W. S. Green P. E. Young M. R. (1996). Hierarchical Bayes conjoint analysis: recovery of partworth heterogeneity from reduced experimental designs. *Mark. Sci.* 15 173–191. 10.1287/mksc.15.2.173 19642375

[B83] Leszczyc P. T. P. Bass F. M. (1998). Determining the effects of observed and unobserved heterogeneity on consumer brand choice. *Appl. Stochast. Models Data Anal.* 14 95–115.

[B84] Liu D. Santhanam R. Webster J. (2017). Toward Meaningful Engagement: a framework for design and research of Gamified information systems. *MIS Q.* 41 1011–1034. 10.25300/misq/2017/41.4.01

[B85] Liu M. Huang Y. Zhang D. (2018). Gamification’s impact on manufacturing: enhancing job motivation, satisfaction and operational performance with smartphone-based gamified job design. *Hum. Fact. Ergon. Manuf. Serv. Ind.* 28 38–51. 10.1002/hfm.20723

[B86] Loch K. D. Straub D. W. Kamel S. (2003). Diffusing the Internet in the Arab world: the role of social norms and technological culturation. *IEEE Trans. Eng. Manage.* 50 45–63. 10.1109/tem.2002.808257

[B87] Locke E. A. Latham G. P. (2002). Building a practically useful theory of goal setting and task motivation: a 35-year odyssey. *Am. Psychol.* 57 705–717. 10.1037/0003-066x.57.9.705 12237980

[B88] Lowry P. B. Gaskin J. Moody G. D. (2015). Proposing the multi-motive information systems continuance model (MISC) to better explain end-user system evaluations and continuance intentions. *J. Assoc. Inf. Syst.* 16 515–579. 10.17705/1jais.00403

[B89] Luo X. Zhang Y. Zeng F. Qu Z. (2020). Complementarity and cannibalization of offline-to-online targeting: a field experiment on omnichannel commerce. *Manage. Inf. Syst. Q.* 44 957–982.

[B90] Manning K. C. Bearden W. O. Madden T. J. (1995). Consumer innovativeness and the adoption process. *J. Consum. Psychol.* 4 329–345. 10.1207/s15327663jcp0404_02

[B91] Marakas G. Johnson R. Clay P. F. (2007). The evolving nature of the computer self-efficacy construct: an empirical investigation of measurement construction, validity, reliability and stability over time. *J. Assoc. Inf. Syst.* 8:2.

[B92] McDaniel R. Fanfarelli J. (2016). Building better digital badges: pairing completion logic with psychological factors. *Simul. Gam.* 47 73–102. 10.1177/1046878115627138

[B93] McGuire W. J. (1974). *Psychological Motives and Communication Gratification.* Beverly Hills, CA: Sage.

[B94] Morford Z. H. Witts B. N. Killingsworth K. J. Alavosius M. P. (2014). Gamification: the intersection between behavior analysis and game design technologies. *Behav. Anal.* 37 25–40. 10.1007/s40614-014-0006-1 27274957 PMC4883455

[B95] Moro S. Ramos P. Esmerado J. Jalali S. M. J. (2019). Can we trace back hotel online reviews’ characteristics using gamification features? *Int. J. Inf. Manage.* 44 88–95. 10.1016/j.ijinfomgt.2018.09.015

[B96] Muthén B. O. (1989). Latent variable modeling in heterogeneous populations. *Psychometrika* 54 557–585. 10.1007/bf02296397

[B97] Neal W. D. (1999). Satisfaction is nice, but value drives loyalty. *Mark. Res.* 11 20–23.

[B98] Niranjanamurthy M. Kavyashree N. Jagannath S. Chahar D. (2013). Analysis of e-commerce and m-commerce: advantages, limitations and security issues. *Int. J. Adv. Res. Comput. Commun. Eng.* 2 2360–2370.

[B99] O’Brien H. L. (2010). The influence of hedonic and utilitarian motivations on user engagement: the case of online shopping experiences. *Interact. Comput.* 22 344–352. 10.1016/j.intcom.2010.04.001

[B100] Overby J. W. Lee E.-J. (2006). The effects of utilitarian and hedonic online shopping value on consumer preference and intentions. *J. Bus. Res.* 59 1160–1166. 10.1016/j.jbusres.2006.03.008

[B101] Pace R. Dipace A. (2015). “Game-based learning and lifelong learning for tourist operators,” in *Cultural Tourism in a Digital Era*, ed. Katsoni V. (Cham: Springer), 185–199. 10.1007/978-3-319-15859-4_16

[B102] Parasuraman A. Zeithaml V. A. Malhotra A. (2005). ES-QUAL: a multiple-item scale for assessing electronic service quality. *J. Serv. Res.* 7 213–233. 10.1177/1094670504271156

[B103] Park J. Ha S. (2016). Co-creation of service recovery: utilitarian and hedonic value and post-recovery responses. *J. Retail. Consum. Serv.* 28 310–316. 10.1016/j.jretconser.2015.01.003

[B104] Peltola S. Vainio H. Nieminen M. (2015). “Key factors in developing omnichannel customer experience with finnish retailers,” in *Proceedings of the International Conference on HCI in Business* (Cham: Springer), 335–346. 10.1007/978-3-319-20895-4_31

[B105] Piotrowicz W. Cuthbertson R. (2014). Introduction to the special issue information technology in retail: toward omnichannel retailing. *Int. J. Electron. Commer.* 18 5–16. 10.2753/jec1086-4415180400

[B106] Podsakoff P. M. Mackenzie S. B. Lee J.-Y. Podsakoff N. P. (2003). Common method biases in behavioral research: a critical review of the literature and recommended remedies. *J. Appl. Psychol.* 88 879–903. 10.1037/0021-9010.88.5.879 14516251

[B107] Podsakoff P. M. Mackenzie S. B. Podsakoff N. P. (2012). Sources of method bias in social science research and recommendations on how to control it. *Annu. Rev. Psychol.* 63 539–569. 10.1146/annurev-psych-120710-100452 21838546

[B108] Podsakoff P. M. Organ D. W. (1986). Selfreports in organizational research: problems and prospects. *J. Manage.* 12 531–544. 10.1177/014920638601200408

[B109] Rai A. Patnayakuni R. Seth N. (2006). Firm performance impacts of digitally enabled supply chain integration capabilities. *MIS Q.* 225–246.

[B110] Rodríguez-Torrico P. Cabezudo R. S. J. San-Martín S. (2017). Tell me what they are like and I will tell you where they buy. An analysis of omnichannel consumer behavior. *Comput. Hum. Behav.* 68 465–471. 10.1016/j.chb.2016.11.064

[B111] Rogers E. M. Shoemaker F. F. (1971). *Communication of Innovations: A Cross-Cultural Approach.* New York, NY: Free Press.

[B112] Saghiri S. Wilding R. Mena C. Bourlakis M. (2017). Toward a three-dimensional framework for omni-channel. *J. Bus. Res.* 77 53–67. 10.1016/j.jbusres.2017.03.025

[B113] Sánchez-Prieto J. C. Olmos-Migueláñez S. García-Peñalvo F. J. (2017). MLearning and pre-service teachers: an assessment of the behavioral intention using an expanded TAM model. *Comput. Hum. Behav.* 72 644–654. 10.1016/j.chb.2016.09.061

[B114] Sarstedt M. Henseler J. Ringle C. M. (2011). “Multigroup analysis in partial least squares (PLS) path modeling: alternative methods and empirical results,” in *Measurement and Research Methods in International Marketing*, eds Sarstedt M. Schwaiger M. Taylor C. R. (Bingley: Emerald Group Publishing Limited), 195–218. 10.1108/s1474-7979(2011)0000022012

[B115] Sarstedt M. Ringle C. M. Cheah J.-H. Ting H. Moisescu O. I. Radomir L. (2019). Structural model robustness checks in PLS-SEM. *Tourism Econ.* 26, 531–554. 10.1177/1354816618823921

[B116] Sheth J. N. Newman B. I. Gross B. L. (1991). Why we buy what we buy: a theory of consumption values. *J. Bus. Res.* 22 159–170. 10.1016/0148-2963(91)90050-8

[B117] Späth H. (1979). Algorithm 39 clusterwise linear regression. *Computing* 22 367–373. 10.1007/bf02265317

[B118] Srite M. Karahanna E. (2006). The role of espoused national cultural values in technology acceptance. *MIS Q.* 679–704.

[B119] Suh A. Cheung C. M. Ahuja M. Wagner C. (2017). Gamification in the workplace: the central role of the aesthetic experience. *J. Manage. Inf. Syst.* 34 268–305. 10.1080/07421222.2017.1297642

[B120] Suh A. Wagner C. (2017). How gamification of an enterprise collaboration system increases knowledge contribution: an affordance approach. *J. Knowl. Manage.* 21 416–431. 10.1108/jkm-10-2016-0429

[B121] Suh A. Wagner C. Liu L. (2018). Enhancing user engagement through gamification. *J. Comput. Inf. Syst.* 58 204–213. 10.1080/08874417.2016.1229143

[B122] To P.-L. Liao C. Lin T.-H. (2007). Shopping motivations on Internet: a study based on utilitarian and hedonic value. *Technovation* 27 774–787. 10.1016/j.technovation.2007.01.001

[B123] Tobon S. Ruiz-Alba J. L. García-Madariaga J. (2019). Gamification and online consumer decisions: Is the game over? *Decis. Support Syst.* 128:113167 10.1016/j.dss.2019.113167

[B124] Toda A. M. Do Carmo R. M. Da Silva A. P. Bittencourt I. I. Isotani S. (2019). An approach for planning and deploying gamification concepts with social networks within educational contexts. *Int. J. Inf. Manage.* 46 294–303. 10.1016/j.ijinfomgt.2018.10.001

[B125] Van Birgelen M. De Jong A. De Ruyter K. (2006). Multi-channel service retailing: the effects of channel performance satisfaction on behavioral intentions. *J. Retail.* 82 367–377. 10.1016/j.jretai.2006.08.010

[B126] Van der Heijden H. (2004). User acceptance of hedonic information systems. *MIS Q.* 695–704.

[B127] Venkatesh V. Morris M. G. Davis G. B. Davis F. D. (2003). User acceptance of information technology: toward a unified view. *MIS Q.* 425–478.

[B128] Verhoef P. C. Kannan P. K. Inman J. J. (2015). From multi-channel retailing to omni-channel retailing: introduction to the special issue on multi-channel retailing. *J. Retail.* 91 174–181. 10.1016/j.jretai.2015.02.005

[B129] Wedel M. DeSarbo W. S. (1994). “A review of latent class regression models and their applications,” in *Advanced Methods for Marketing Research*, ed. Bagozzi R. P. (Cambridge, MA: Blackwell), 353–388.

[B130] Wee C.-H. Ta S.-J. Cheok K.-H. (1995). Non-price determinants of intention to purchase counterfeit goods: an exploratory study. *Int. Mark. Rev.* 12 19–46. 10.1108/02651339510102949

[B131] Wu T. Seidmann A. (2018). Can irrelevant benchmark information help when making business decisions under uncertainty? An empirical investigation of the newsvendor game. *Decis. Support Syst.* 107 40–51. 10.1016/j.dss.2017.12.014

[B132] Xi N. Hamari J. (2019). Does gamification satisfy needs? A study on the relationship between gamification features and intrinsic need satisfaction. *Int. J. Inf. Manage.* 46 210–221. 10.1016/j.ijinfomgt.2018.12.002

[B133] Yoo C. W. Sanders G. L. Moon J. (2013). Exploring the effect of e-WOM participation on e-Loyalty in e-commerce. *Decis. Support Syst.* 55 669–678.

[B134] Yurova Y. Rippé C. B. Weisfeld-Spolter S. Sussan F. Arndt A. (2017). Not all adaptive selling to omni-consumers is influential: the moderating effect of product type. *J. Retail. Consum. Serv.* 34 271–277. 10.1016/j.jretconser.2016.01.009

